# Design Considerations, Field Validation and Perspectives of a Low-Power Wearable Sensor Collar for Continuous Monitoring of Ruminants

**DOI:** 10.3390/s26165019

**Published:** 2026-08-07

**Authors:** Maria P. Nikolopoulou, Aikaterini-Artemis Agiomavriti, Dimitrios Loukatos, Dimitrios Grivas, Nikolaos Xotzempekoglou, Athanasios I. Gelasakis, Konstantinos G. Arvanitis, Konstantinos Demestichas, Thomas Bartzanas

**Affiliations:** 1 Laboratory of Farm Structures, Department of Natural Resources Management & Agricultural Engineering, Agricultural University of Athens, Iera Odos 75 str., 11855 Athens, Greece or mnikolopoulou@tcb.gr (M.P.N.); t.bartzanas@aua.gr (T.B.); 2 R&D Department, TCB Avgidis Automation S.A., 11744 Athens, Greece or agiomavriti@aua.gr (A.-A.A.); dgrivas@tcb.gr (D.G.); nxotzempekoglou@tcb.gr (N.X.); 3 Laboratory of Anatomy and Physiology of Farm Animals, Department of Animal Science, School of Animal Biosciences, Agricultural University of Athens, Iera Odos 75 str., 11855 Athens, Greece; gelasakis@aua.gr; 4 Laboratory of Farm Machine Systems, Department of Natural Resources Management & Agricultural Engineering, Agricultural University of Athens, Iera Odos 75 str., 11855 Athens, Greece; karvan@aua.gr; 5 Laboratory of Informatics, Department of Agricultural Economics and Rural Development, Agricultural University of Athens, Iera Odos 75 str., 11855 Athens, Greece; cdemest@aua.gr

**Keywords:** livestock monitoring, wearables, collars, on-site technologies, wireless sensors, physical computing, performance evaluation, smart agriculture

## Abstract

Wearable sensors provide significant potential for continuous animal monitoring. However, the practical implementation of such technologies on livestock farms for animal monitoring raises significant challenges related to power efficiency, robustness, and reliability. In this research paper, we propose the design, implementation, and field testing of a low-energy, low-cost, multi-sensor wearable collar specifically designed for continuous monitoring of ruminants using LoRa. The proposed collar is based on a modular hardware platform that incorporates inertial sensing, temperature sensing, and wireless communication, with special attention to sensor choice, location on the body, casing, and mounting mechanism. Reduced weight, environmental protection, and long-term wearability without affecting animal behavior are the primary focus in the hardware design. Power optimization management strategies, including sleep mode and duty cycling functionality, are implemented to maximize autonomy and battery lifetime and are evaluated under realistic operating scenarios. Field deployment was conducted in a ruminant farm, where the wearable devices operated flawlessly for a long time period. Characteristic sensor data are collected, including accelerometer readings induced by animal movement, variability in received signal strength (RSSI), and animal temperature. The system demonstrates stable operation, satisfactory data completeness and consistency on multi-day basis. The knowledge acquired brings into focus the practical challenges and design issues associated with the use of wearable sensors in livestock and offers insights into designing efficient sensor collars for precision livestock farming.

## 1. Introduction

Livestock farming is rapidly transforming into data-driven and technology-assisted management systems. Producers’ main aim is to improve productivity while maintaining high standards of animal welfare [1]. Ensuring animals’ wellbeing is crucial for both ethical and financial reasons. In this context, digital technologies offer continuous monitoring of animal behavior and their health indicators, enabling early detection of health problems and prompt treatment [2]. Traditional livestock monitoring still relies mainly on visual inspection and routine manual checks. This need is reinforced by the broader precision livestock farming (PLF) concept, where real-time monitoring is intended to support the farmer before health or welfare problems become severe [3,4]. In their study, Berckmans [5] emphasized that PLF should provide early warnings during the rearing period, rather than identifying problems only after delayed observation or post-event assessment. These methods are subjective, labor-intensive, and can usually lead to human mistakes. These issues are especially evident when trying to identify conditions like lameness, where early signs are subtle and frequently missed. Standard visual assessment methods lack reliability and may delay treatment, resulting in lower productivity and negatively impacting on animal welfare [6,7].

Precision livestock farming aims to address and reduce these challenges by using wearable sensing technologies, connectivity, and data-driven analysis [8]. Among these, the use of collar-mounted devices has arisen as one of the most effective and widely adopted solutions, since they can host multiple sensing modalities while remaining non-invasive and suitable for continuous use on animals [9,10]. Inertial sensing, specifically, offers vital information about animal movement and behavior, making the identification of patterns associated with locomotion, feeding, resting, and various other activities easier and more precise. Changes in these movement patterns may indicate alterations in animal behavior and welfare, providing valuable information for continuous livestock monitoring [6,11]. This is consistent with studies in sheep showing that accelerometers and GPS-based monitoring can detect changes in movement and behavior, while real-time or near-real-time transmission may allow faster farmer response when animals become ill [12,13].

The key advantage of wearable systems over non-contact monitoring methods is the potential for continuous monitoring and individual-level data collection in both indoor and outdoor environments. Inertial sensors, such as accelerometers and gyroscopes, are valuable because they register three-dimensional movement and posture changes associated with lying, standing, walking, feeding and rumination [14,15]. However, their performance is highly dependent on the sensor location, the sampling frequency, attachment stability and the selected data processing procedure. The engineering design of the wearable device and the analytical treatment of the recorded signals should be regarded as overlapping processes rather than isolated steps [16]. This integrated view is further supported by recent end-to-end livestock monitoring frameworks, which emphasize that the sensor hardware, data acquisition, feature extraction and health assessment should be designed as a unified workflow rather than independent components [16].

The latest advancements further highlight the significance of integrating multiple sensing modalities into wearables to enhance the interpretation of behavior since the integration of the information related to movements with other parameters like temperature and communication enables a more comprehensive assessment of animal condition without increasing system complexity [17]. Notably, the use of such devices in livestock farming is determined by their ability to function effectively in actual environmental settings, which calls for field tests and on-site measurements [18]. In fact, recent livestock research has stressed the significance of validating sensing technologies in real-life conditions, with measurements at the field level becoming indispensable to determine their reliability, reproducibility, and practicality before large-scale adoption [19]. Studies have demonstrated the potential of wearable sensing systems for livestock monitoring by using accelerometers, gyroscopes and wireless communication modules. Inertial measurement units (IMUs) have been successfully used for activity recognition, including walking, grazing, and resting, while machine learning techniques have further expanded their application to automated behavior analysis and health monitoring [2,5]. Recently published reviews emphasize the growing role of collar-mounted sensors in PLF and their ability to monitor behaviors continuously. However, almost all of the proposed approaches concentrate mainly on classification and were usually tested under controlled conditions or only for a short period of time. Hence, the problems of reliability, energy autonomy, practical wearability, and usability under commercial livestock conditions remain insufficiently addressed [4].

Moreover, most of the published studies focus on either sensor performance, communication technologies or data analysis separately while only a few of them consider the overall engineering design of wearable collars including sensor integration, power management, enclosure development, attachment strategy and long-term field validation in a single platform [20]. Thus, practical design trade-offs that determine whether a prototype can operate reliably under commercial livestock conditions are poorly documented [21]. To close this gap, the systematic validation of sensing technologies as complete wearable systems operating in realistic farm environments is required, in addition to the development of sensing technologies.

Such systems depend heavily on low power wireless communication technologies. Long-range communication and low energy consumption are important in rural and large-scale farm environments. LoRa-based communication has been proven to be a suitable solution, providing reliable long-range data transmission and allowing energy-efficient operation thanks to duty-cycling and sleep modes [22].

There are, however, several challenges to the practical deployment of wearable systems in livestock environments. Devices have to work under tough conditions such as dust, moisture, and the mechanical stress of animal movement. They should be lightweight, unobtrusive and able to operate for long periods with limited energy resources. Therefore, power consumption, communication reliability and mechanical robustness are important design constraints [9]. In addition, wearable devices deployed on animals are subject to continuous motion, impacts, and variable environmental conditions that may affect sensor stability and data quality. Achieving reliable sensor positioning, maintaining user comfort, minimizing maintenance requirements, and ensuring long-term battery autonomy represent additional challenges that complicate large-scale adoption in commercial farms. Moreover, the usage of wearable devices on animals involves continuous movement, impacts and variations in the environment that might compromise sensor stability and the quality of data. Additional challenges that hinder large-scale adoption in farms include ensuring reliable sensor positioning, user comfort, low maintenance requirements, and long-term battery autonomy. However, there is a significant difference between experimental prototypes and systems which can work reliably in real farm conditions for long periods. Another related limitation is that many published systems focus on a single isolated stage such as hardware development, behavior classification or model optimization without considering the continuity of the full monitoring process. This fragmentation hampers practical implementation since field applications call for stable sensing, standardized data formatting, robust communication, interpretable variables and clear presentation of results all within the same operational context. Recent studies have thus focused on modular and end-to-end architectures that connect wearable sensing with structured processing and decision-oriented outputs [16]. Many existing studies focus on controlled environments or short-term deployments [23].

Besides sensor integration, the current trends in PLF are geared towards practical and scalable wearable solutions that can operate autonomously for extended periods of time in real farm conditions. Innovation is therefore moving from proof-of-concept devices to rugged systems with multi-sensor data acquisition, low-power electronics, optimized communication strategies and ergonomic mechanical design. There is a need for such integrative approaches in order to transfer research prototypes to deployable technologies in routine livestock management.

Therefore, the objective of this pilot proof-of-concept study was to evaluate the feasibility and field performance of a wearable collar system for monitoring cattle under commercial farming conditions. The proposed system addresses practical engineering aspects including sensor selection, sensor placement, enclosure design, attachment method, and power optimization. Special focus is on long-term deployment, robustness of the system and energy-efficient operation in real farm conditions. The study further explores the extraction of meaningful behavioral indicators from synchronized multi-sensor measurements and provides experimentally validated design guidelines for wearable livestock devices. Although recent studies have reported important advances in wearable livestock monitoring, comparatively fewer publications provide a detailed engineering analysis of the complete design process, including hardware integration, power management, communication architecture, enclosure development, and long-term validation under practical farm conditions. The present work addresses this gap by documenting the engineering design decisions and performance trade-offs associated with the development of a low-cost wearable collar intended for reliable long-term deployment in ruminant production systems. In addition to presenting the final system, the study reports the practical experience gained during its iterative development and field validation, highlighting both successful engineering solutions and implementation approaches that proved less effective after experimental evaluation. We believe that these practical aspects are often underreported in the literature, despite being highly relevant for researchers seeking to translate laboratory prototypes into reliable field-deployable systems. By documenting these design choices and lessons learned, the manuscript aims to provide practical guidance for researchers and engineers developing future wearable livestock monitoring systems.

As shown in Table 1, while recent studies have primarily focused on sensing performance, behavior recognition, or communication technologies, comparatively fewer publications provide a comprehensive description of the engineering design process, power optimization, mechanical implementation, and long-term field validation of wearable livestock monitoring systems.

## 2. Materials and Methods

The proposed wearable collar was experimentally evaluated at the livestock farm of the Agricultural University of Athens, Greece. The study was conducted on a herd of approximately ten multiparous dairy cows of Frezian Holstein breed, housed under a semi-extensive production system that included daily access to both indoor housing facilities and outdoor exercise areas as part of routine management. The developed wearable collars were fitted on four dairy cows and monitored under normal farm management conditions. The cows were selected randomly as the collars were attached by conventional neck straps and did not interfere with the animals’ routine activities, so the system could be tested under realistic operational conditions.

The wearable collar system was field-tested for approximately three months to evaluate the long-term operational stability and robustness. Continuous measurements have been acquired over the whole deployment, while the dataset analyzed in the present study refers to a representative operation of about one month. Over this period collars continuously sampled inertial measurements, collar temperature, battery status and communication-related information. The data were transmitted to the gateway through LoRa network and stored for later processing and analysis. This deployment made it possible to evaluate the proposed system under the environmental conditions and operational challenges that are typical in semi-extensive livestock farming.

### 2.1. Collar System Architecture

**Figure 1:**
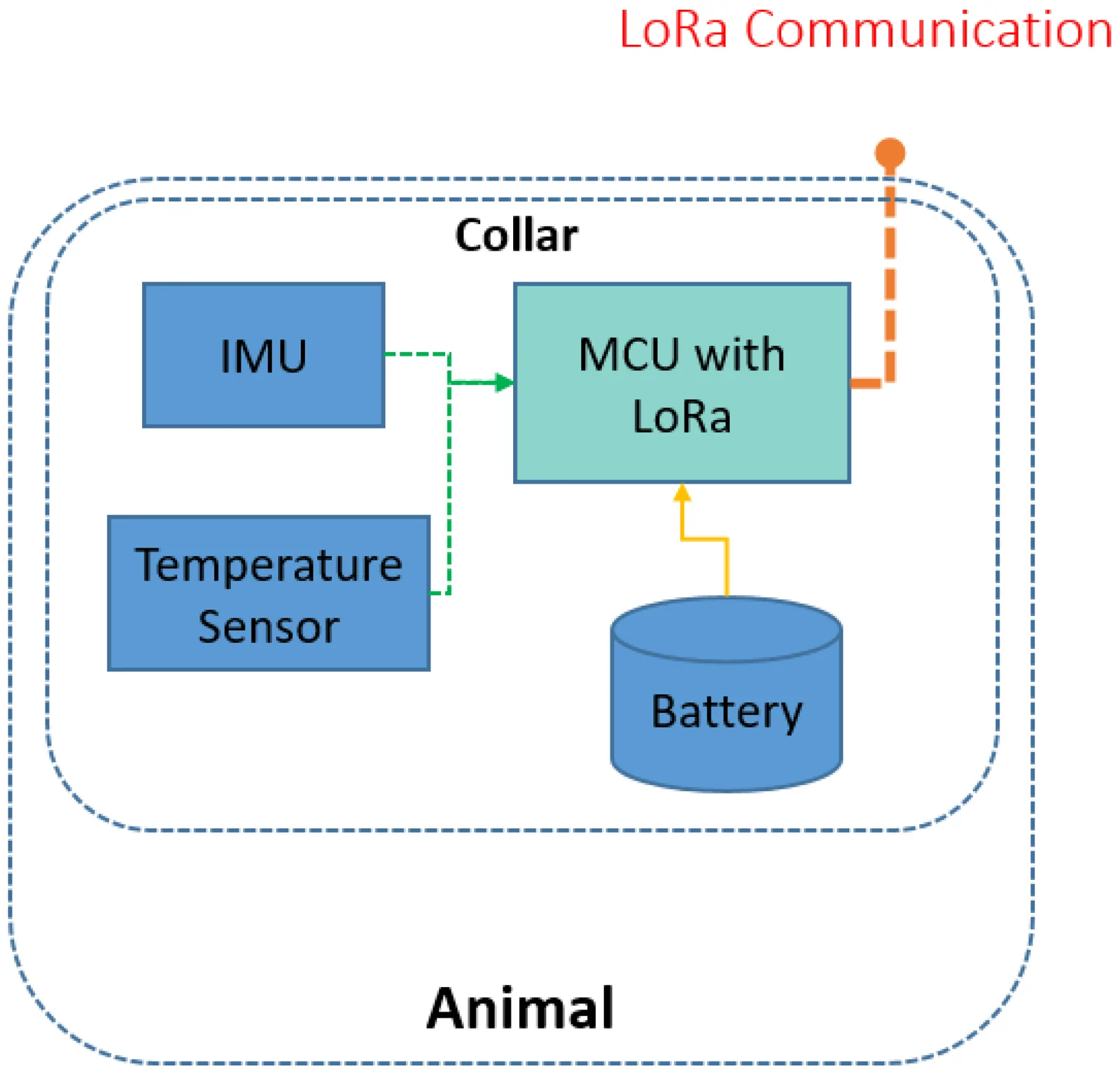
Block diagram of the wearable collar, including sensing modules, microcontroller, power management, and LoRa communication.

The proposed system is based on a wearable collar platform designed for continuous monitoring of individual animals under real-farm conditions. The overall aim was to develop a low-power collar-based platform which can collect data from different animals and transmit these data to a remote server infrastructure. The system architecture was thus divided into two main device roles, namely sender collars and a receiver or gateway node. The overall design and implementation principles are following the guidelines as described in Loukatos et al. [22]. The sender collars collected animal-specific measurements and wirelessly transmitted the information. The receiver device was collecting the transmitted information and sending it to the cloud. Each collar operates as an autonomous sensing node integrating sensing, processing, power management, and wireless communication capabilities within a compact and robust enclosure. The hardware architecture of the wearable collar is presented in Figure 1. LoRa communication was used for the local farm-level wireless layer due to its long-range transmission with low power consumption. This becomes particularly important in livestock settings where animals may roam over large outdoor areas and conventional short-range technologies, such as Wi-Fi or Bluetooth, are not sufficient. In the proposed topology, multiple sender collars are worn by individual animals distributed throughout the herd, while a single receiver node collects the data packets transmitted by each collar. This architecture reduces the need for each collar to maintain a direct Internet connection, thereby reducing energy consumption, hardware complexity and communication cost at the animal-node level.

The collar hardware is built around a low-cost microcontroller, which combines embedded low-power processing capabilities with long-range wireless communication, based on the Atmel 32u4, running at 8 MHz. Initially, an Adafruit Feather 32u4 RFM95 LoRa Radio board was utilized, while the deployment also benefited from the LoRa32u4 II development board, which had an attractive price and a U.FL coaxial connector socket and cable for hosting standardized LoRa antennas. This board implements a very efficient sleep-mode function and has a compact form factor, making it suitable for fitting inside a wearable collar.

The sensing subsystem includes an IMU used to capture motion-related data, as well as a temperature sensor for basic physiological monitoring. The thermometer was implemented using a TMP26 chip, which is capable of low voltage operation. These sensors were selected based on their low power consumption, compact size, and suitability for continuous operation in livestock environments.

The temperature sensor was embedded within a recessed cavity of the collar enclosure and thermally coupled to the animal through a metallic screw in direct contact with the skin. This configuration was designed to improve thermal coupling with the animal while protecting the sensor from mechanical damage. Consequently, the recorded measurements represent a surface-contact temperature influenced by both the animal’s body surface and the surrounding environmental conditions. Therefore, the measurements should be regarded as a proxy of the local body-surface thermal state rather than a direct measurement of core body temperature.

Sensor placement on the animal was carefully considered to ensure reliable data acquisition while minimizing interference with their natural behavior. The collar was positioned around the neck, allowing stable measurement of movement patterns without restricting the animal’s mobility. Collected data are transmitted from the device to a nearby base station using LoRa communication. This approach enables long-range communication with low energy consumption, making it suitable for deployment in large farm environments where animals may move over extended areas.

### 2.2. Powering and Mechanical Design Requirements

**Figure 2:**
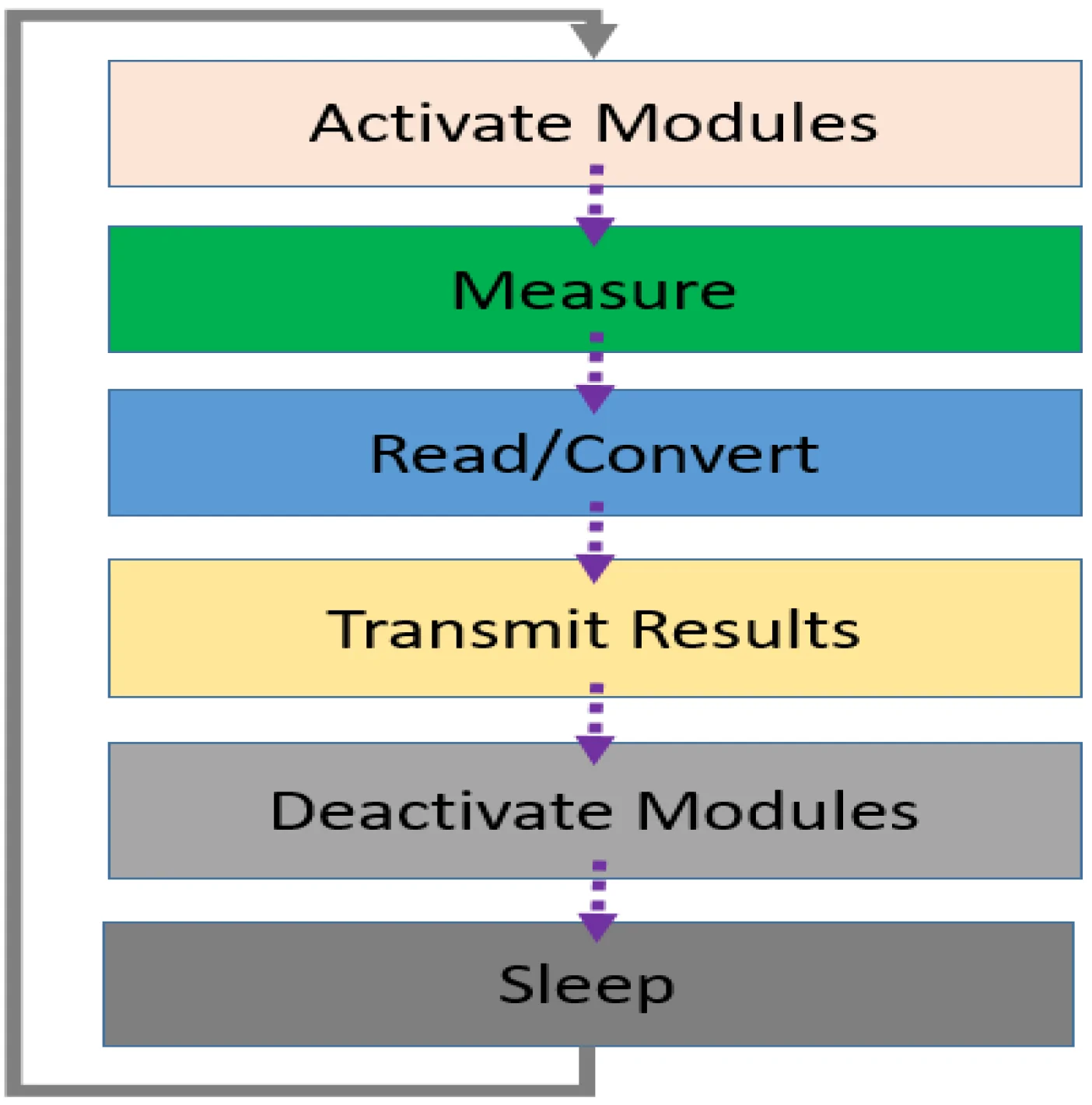
Sequence of the basic functions comprising the logic of the collars.

Power management is a critical design aspect of the wearable collar since the system needs to operate continuously for long periods of time with limited energy resources. The device is powered by lithium-based batteries, chosen for their capacity, weight and operational stability. In the deployed configuration, the device follows a duty-cycling strategy, alternating between sensing and transmission phases and low-power sleep periods. This allows a significant reduction in the average energy consumption and multi-day autonomous operation without loss of the ability to capture representative activity patterns. To better meet these requirements, an activity period of 13 s, containing sensing, processing and transmit actions, is followed by a suitable sleep period, to achieve periodic data delivery every 2.5 min, approximately. The sequence of the basic functions comprising the logic of the collars is depicted in Figure 2.

**Figure 3:**
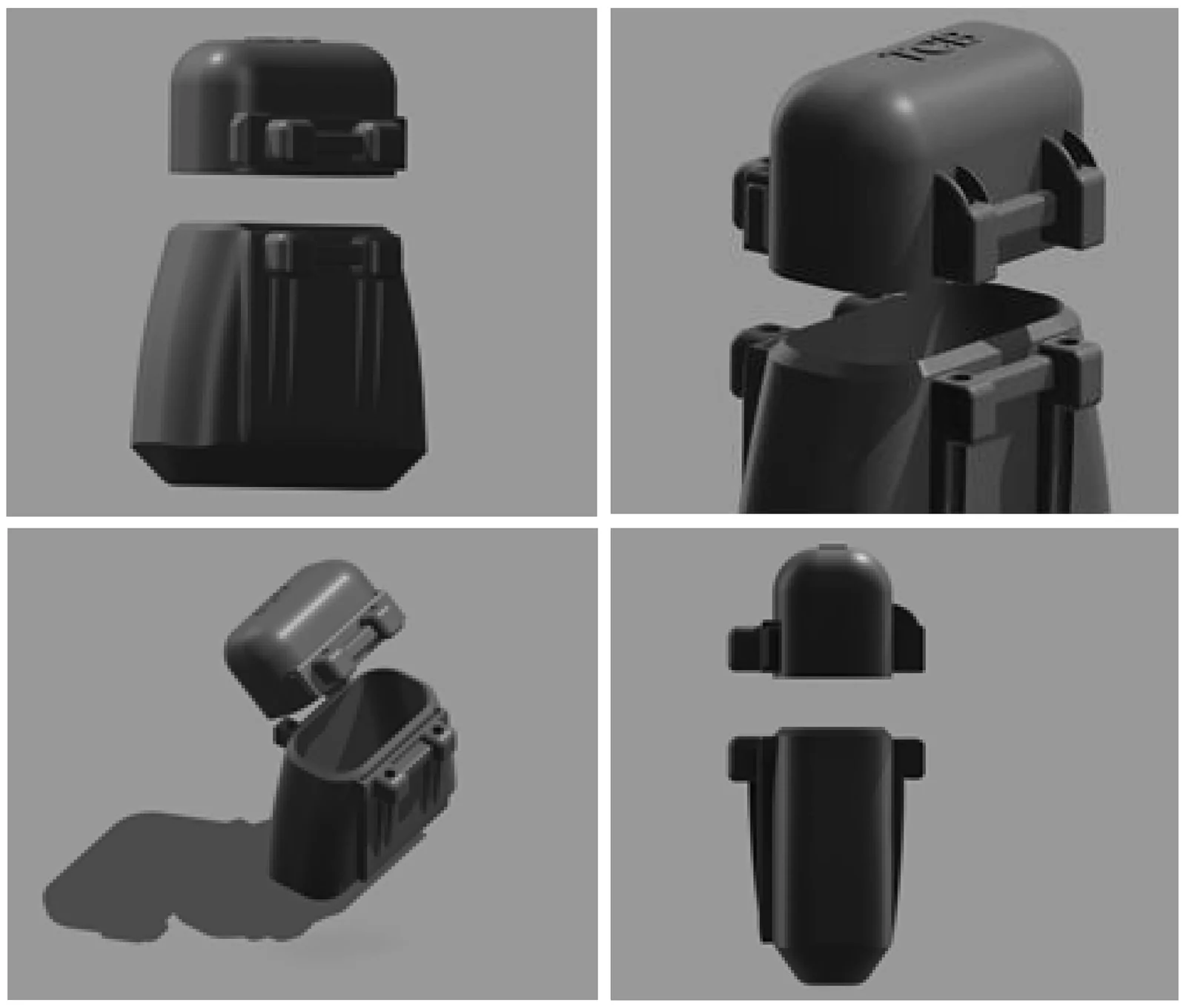
Custom-designed enclosure for the wearable collar, optimized for durability and animal comfort.

Mechanical design is equally important for ensuring reliable operation in real farm conditions. The enclosure was designed to protect electronic components from dust, moisture, and mechanical impacts, while remaining lightweight and unobtrusive for the animal. The housing was manufactured using 3D printing, allowing rapid prototyping and iterative design improvements. The final enclosure design is presented in Figure 3.

The attachment method was designed to ensure stable positioning of the device without causing discomfort or restricting movement. Field testing confirmed that the collar can be worn for extended periods without affecting normal animal behavior.

### 2.3. Data Acquisition, Communication and Processing

**Figure 4:**
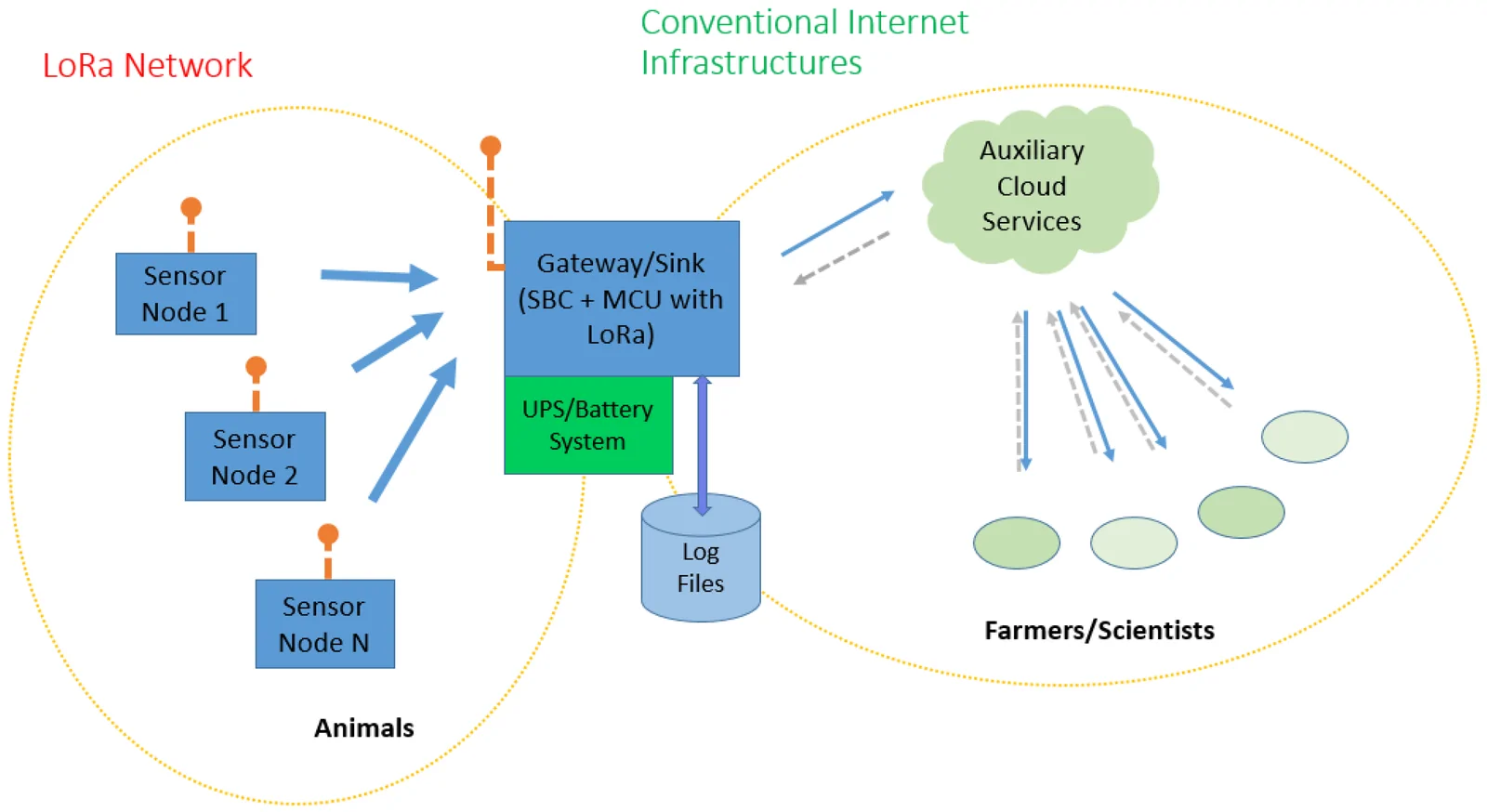
Explanation of the method according to which the information coming from the collars is gathered and made accessible to the cloud via a suitable edge node.

The wearable collar continuously collects data related to animal activity, temperature, battery status, and communication signal characteristics. Motion data are acquired through the IMU and processed locally to extract basic statistical features over predefined time intervals. Data transmission is performed using LoRa communication, allowing reliable long-range connectivity with low energy consumption. The collar periodically transmits processed data packets to a base station, where they are stored and made available for further analysis. A typical collar connection scheme with the rest of the platform is illustrated in Figure 4.

All collars are sending data in real time, via the LoRa protocol, to a sink/gateway node implemented using a Raspberry Pi (RPi) single board computer (SBC), for further processing and storage, and to make them widely available via a common network infrastructure [26]. The RPi board is equipped with a LoRa receiver module having an omnidirectional antenna of 5 dBi gain, suitable for the 868/915 MHz bands. To improve reliability and minimize data losses the gateway node was also equipped with an Uninterruptible Power Supply (UPS) unit tailored for RPi and a dedicated accurate hardware real-time clock (RTC) unit. In alternative configuration settings, a Raspberry Pico equipped with a LoRa receiver and a 3G modem was also tested, forming a lighter and more portable scheme.

During each acquisition cycle, the collar collected 32 consecutive samples from the temperature sensor, battery-monitoring circuit, accelerometer, and gyroscope. Samples were separated by approximately 328 ms, resulting in a measurement window of about 10.5 s. For temperature and battery voltage, the arithmetic mean of the 32 samples was calculated. For the accelerometer, the mean value of each axis was calculated as $a_{x,avg},\;a_{y,avg},\;and\;a_{z,avg}.$ The minimum and maximum observed values of each acceleration axis were also retained and combined into vector-magnitude metrics according to

(1) $$a_{min}=\sqrt{a_{x,min}^{2}+a_{y,min}^{2}+a_{z,min}^{2}}$$

and

(2) $$a_{max}=\sqrt{a_{x,max}^{2}+a_{y,max}^{2}+a_{z,max}^{2}}$$

The same procedure was applied to the gyroscope measurements, producing the mean angular-velocity values $g_{x,avg},\;g_{y,avg},\;and\;g_{z,avg}$, together with the vector-magnitude metrics $g_{min}\;and\;g_{max}.$ The transmitted packet therefore contained the animal identifier, mean battery voltage, mean collar temperature, three mean acceleration components, minimum and maximum acceleration magnitudes, three mean gyroscope components, and minimum and maximum gyroscope magnitudes. This lightweight feature extraction strategy was intentionally selected to minimize computational load, memory usage, transmission payload, and energy consumption, thereby supporting long-term autonomous operation within the hardware constraints of the selected embedded platform. It must be noted that the microcontroller unit being used, hosting the Atmel 32u4 chip, has a very efficient and thoroughly tested active/sleep mode behavior, but is comparatively slow as it runs at 8 MHz and also has only 2 KB of main random access memory (RAM), thus limiting the options for sophisticated on-device processing and for executing space-demanding AI models.

Each acquisition window lasted approximately 11.0 s, after which the summarized metrics (full list in Table 2) were transmitted through the LoRa link, while a small random delay was added to minimize potential repetitive data packet collisions between two collars [27]. The device then entered a low-power sleep period before the next acquisition cycle [28]. The gateway appended the received signal strength indicator (RSSI) to each stored record.

To ensure data reliability, a local buffering mechanism is implemented. In cases of temporary communication loss, data are stored locally and transmitted once connectivity is restored. This approach guarantees data continuity and prevents information loss under real farm conditions. In addition to basic data acquisition, the system implements a lightweight processing strategy to derive meaningful behavioral information. A lightweight behavioral indicator is defined by combining motion-related features derived from accelerometer data with communication signal characteristics, such as RSSI values. This approach enables the extraction of activity and relative movement patterns directly at the device level, without requiring additional localization hardware or complex processing.

Variations in the accelerometer signals can provide information on activity levels, and variations in RSSI can give indirect indications of relative movement in the farm environment. This combination of signals provides a more complete interpretation of animal behavior without the need for additional hardware or an increase in system complexity.

### 2.4. Statistical and Correlation Analysis

The sensor records were imported and processed using the R statistical computing environment. Date and time fields were combined into a single timestamp using the lubridate package, and records with invalid or unparsable timestamps were removed before analysis. Data manipulation was performed using functions from the tidyverse collection of packages.

Separate Pearson correlation matrices were calculated for each animal to explore linear associations among battery voltage, surface-contact temperature, RSSI, mean acceleration along the X, Y, and Z axes, minimum and maximum acceleration magnitude, mean angular velocity along the X, Y, and Z axes, and minimum and maximum angular-velocity magnitude. Missing values were handled using pairwise complete observations. The correlation matrices were visualized using the ggcorr() function of the GGally package.

A separate exploratory analysis was performed to explore the relationship between the RSSI measurements recorded for the four animals. Because collar transmissions were not always recorded at identical timestamps, the RSSI values were aggregated into common five-minute intervals. The mean RSSI value was calculated for each animal within each interval, and Pearson correlation coefficients were computed using the available paired observations. The selected five-minute interval represented a practical compromise between temporal resolution and the availability of synchronized observations across collars.

The correlation analyses were descriptive and exploratory in nature. No formal significance testing or causal interpretation was applied, and the resulting coefficients were used only to identify potential patterns and associations for further investigation.

## 3. Implementation Details

### 3.1. In Situ Component Arrangments

**Figure 5:**
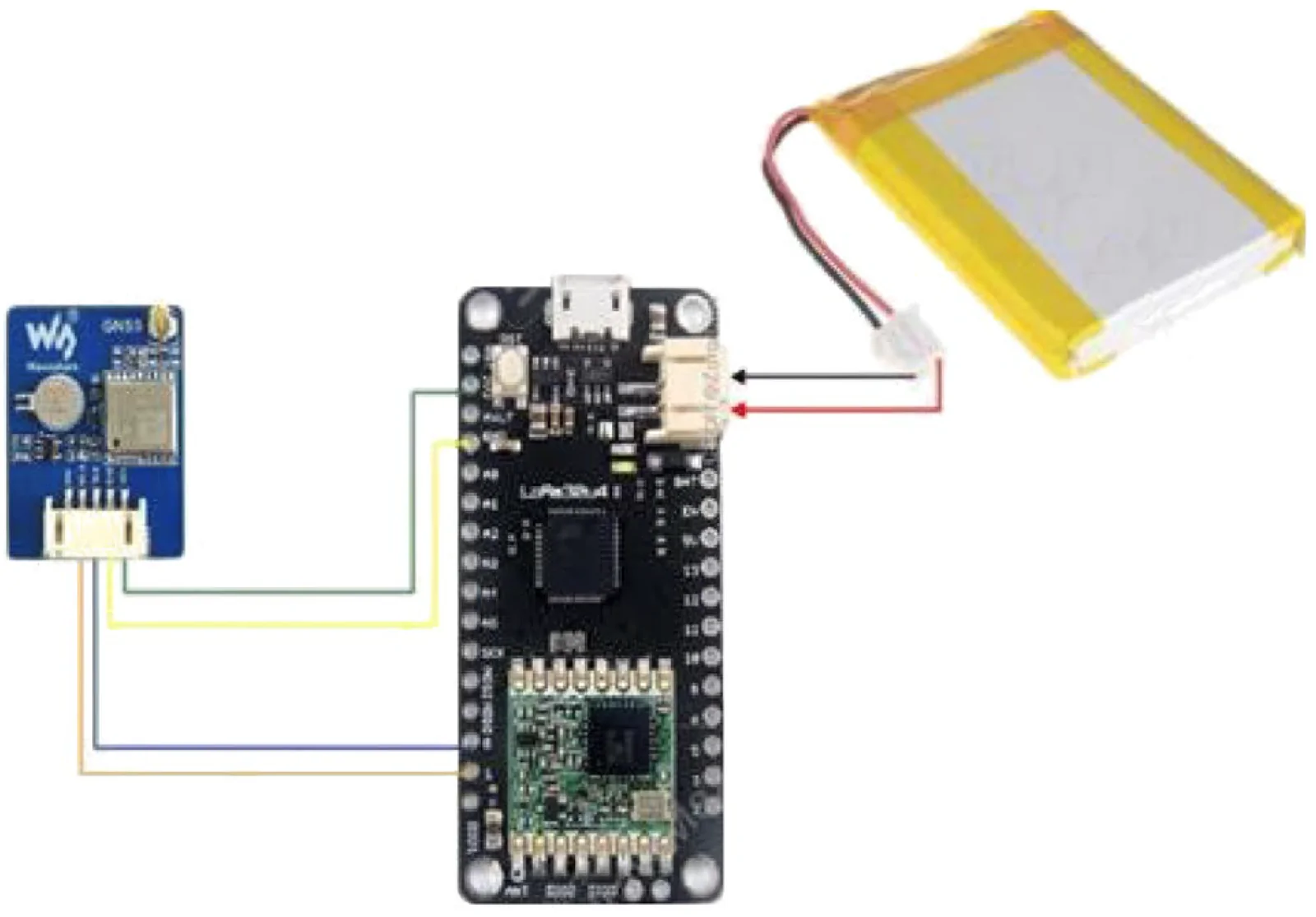
Intermediate hardware prototype transmitter configuration used during system development and evaluation.

Early prototypes revealed constraints in sensor stability, communication reliability, and mechanical robustness, which were overcome through iterative enhancements in component selection, enclosure design, and system integration [29]. Figure 5 shows some representative intermediate hardware configurations developed during this process. These include previous transmitter and receiver implementations used to assess communication performance, power requirements, and system stability. The insights from these intermediate designs inform the development of the final wearable collar architecture. The final hardware configuration was developed iteratively over several stages of design refinement. In further optimization, the data were encrypted using the lightweight Crypt and Speck libraries that are compatible with the Arduino IDE environment.

**Figure 6:**
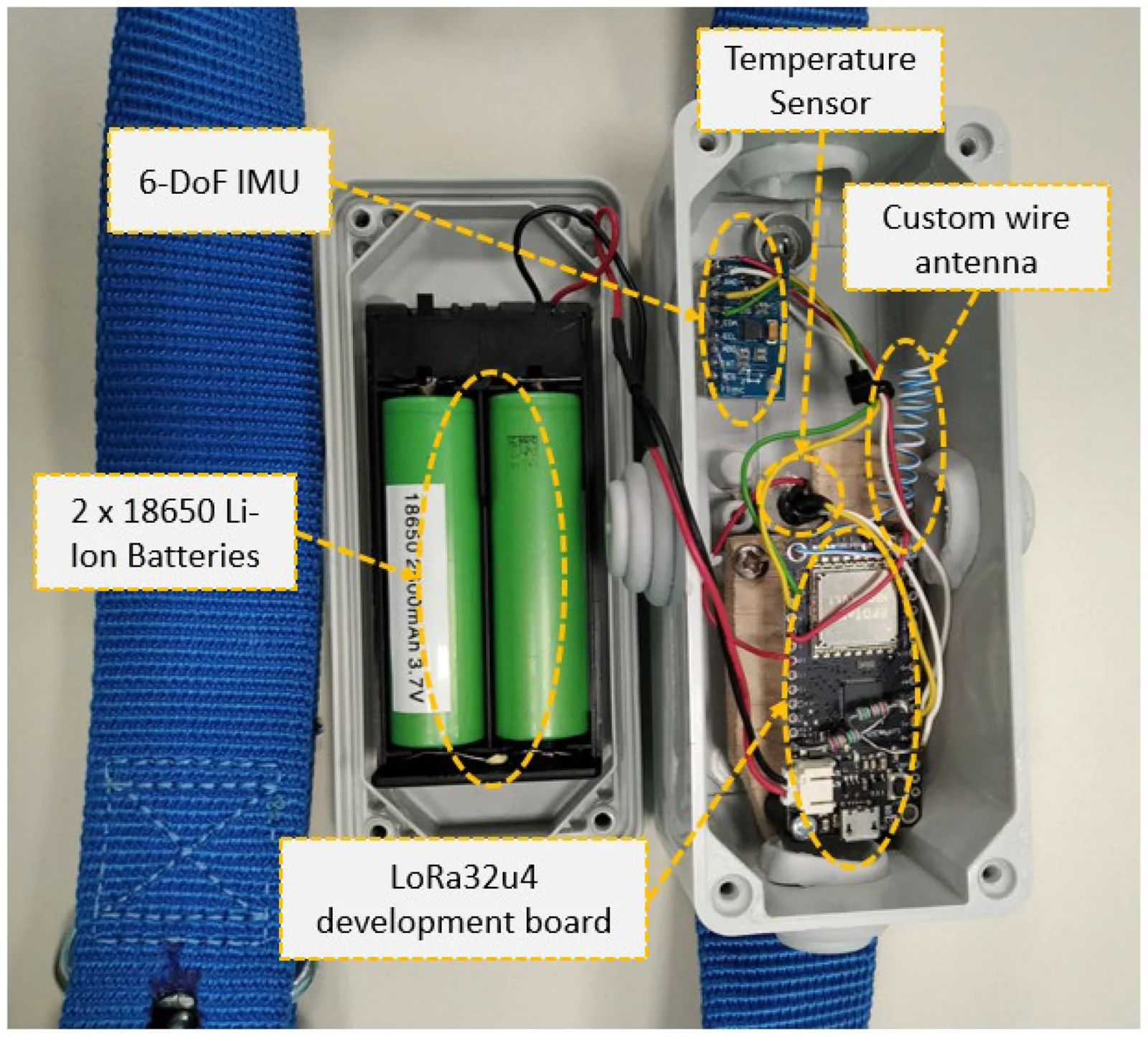
Typical placement of components inside the collar housing.

Figure 6 illustrates a typical arrangement of the components and is placed inside the collar housing during the intermediate series of experiments.

Delving deeper, regarding the engineering characteristics of the proposed collars, initial implementations utilized ready IP65 protection rating plastic box enclosures, similar to the ones used to protect outdoor electric junctions, typically of 55 mm × 13.5 mm × 6.0 mm, in width, length and height, respectively. These boxes were adequate, in terms of size, to host the electronic and powering components of the collars but problems were experienced regarding their fixing on the belts embracing the neck of the animals, while their angular shape and their limited thickness (i.e., of around 1 mm) increased the risk of them snagging on the farm surroundings, becoming detached from the animals and/or being trampled by them. These limitations resulted in designing and utilizing custom 3D printed enclosures of increased thickness (i.e., of around 2–3 mm) and durability, equipped with reinforced holes for passing the belt through them and rounded edges for limiting the risk of damaging them in the farm environment. These collar enclosures were slightly wider, shorter and of lowered profile compared with the aforementioned plastic boxes and comprised two parts that fitted together via bolts and a flange. Initial 3D printed prototypes were made of thermoplastic filament using the Fused Deposition Modeling (FDM) method and Polylactic Acid (PLA) material. As an upgrade, PLA was replaced by Acrylonitrile Butadiene Styrene (ABS) that provides tough and heat-resistant objects. Although ABS is more than adequate, an alternative option was the utilization of polyethylene terephthalate glycol (PETG), which has even better characteristics than the ABS plus flexibility under pressure, to absorb shocks without cracking and to facilitate the sealing of the containing electronics. The only drawback of PETG is that it requires meticulous printer preparation. In all cases, to 3D print the custom housing, approximately 80 g of filament was necessary.

**Figure 7:**
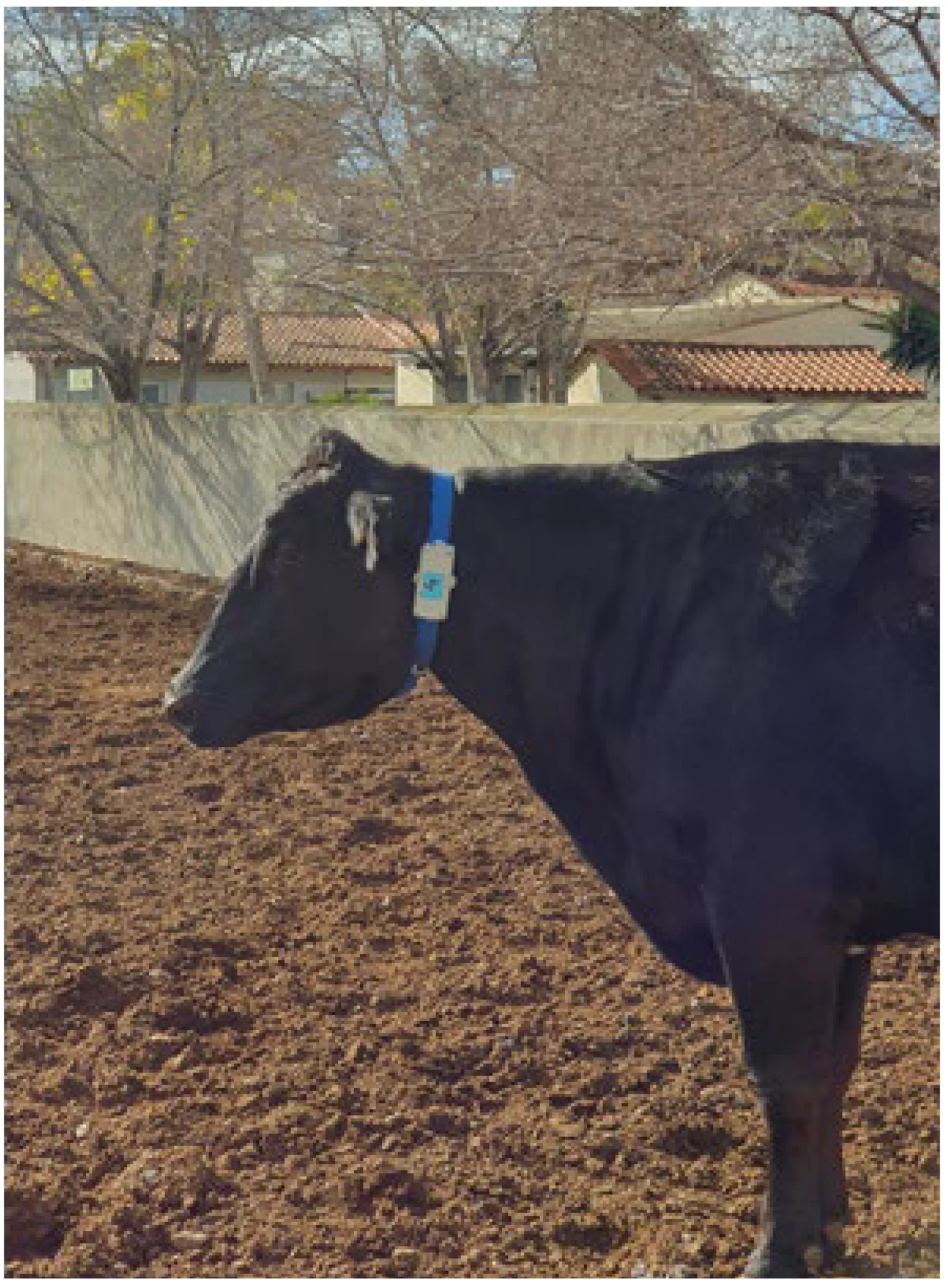
Wearable collar mounted on a cow during field deployment.

Particular attention was given to maintaining consistent orientation of the IMU to ensure the repeatability and interpretability of measurements. A prototype of the wearable collar during field deployment is shown in Figure 7.

In greater detail, regarding the orientation and fixing arrangements of the collars, and with respect to the fact that the differences between periodic readings for a specific animal count more than the corresponding absolute values, it was preferable to follow a uniform installation protocol for all collars placed around the animal necks. More specifically, the enclosures were fixed to the upper left side of the neck the cows. The IMU was fixed inside the upper part of the collar enclosure with the *Y*-axis pointing upwards (toward the sky), the *X*-axis toward the rear of the animal and the *Z*-axis pointing to the left of the animal. The LoRa 32u4 was fixed vertically inside the enclosure with the antenna being also being vertically (longitudinally) placed in parallel with the *Y*-axis. The thermometer unit was placed at the center of the enclosure with its metallic probe facing toward the skin of the animal. It should be noted that, apart from the enclosure itself, a small weight was fixed upon the belt, to counterbalance the weight of the enclosure and of its content, permitting the monitoring system to be kept in place, regardless of the movements of the animal.

### 3.2. Data Accessibility Arrangements

**Figure 8:**
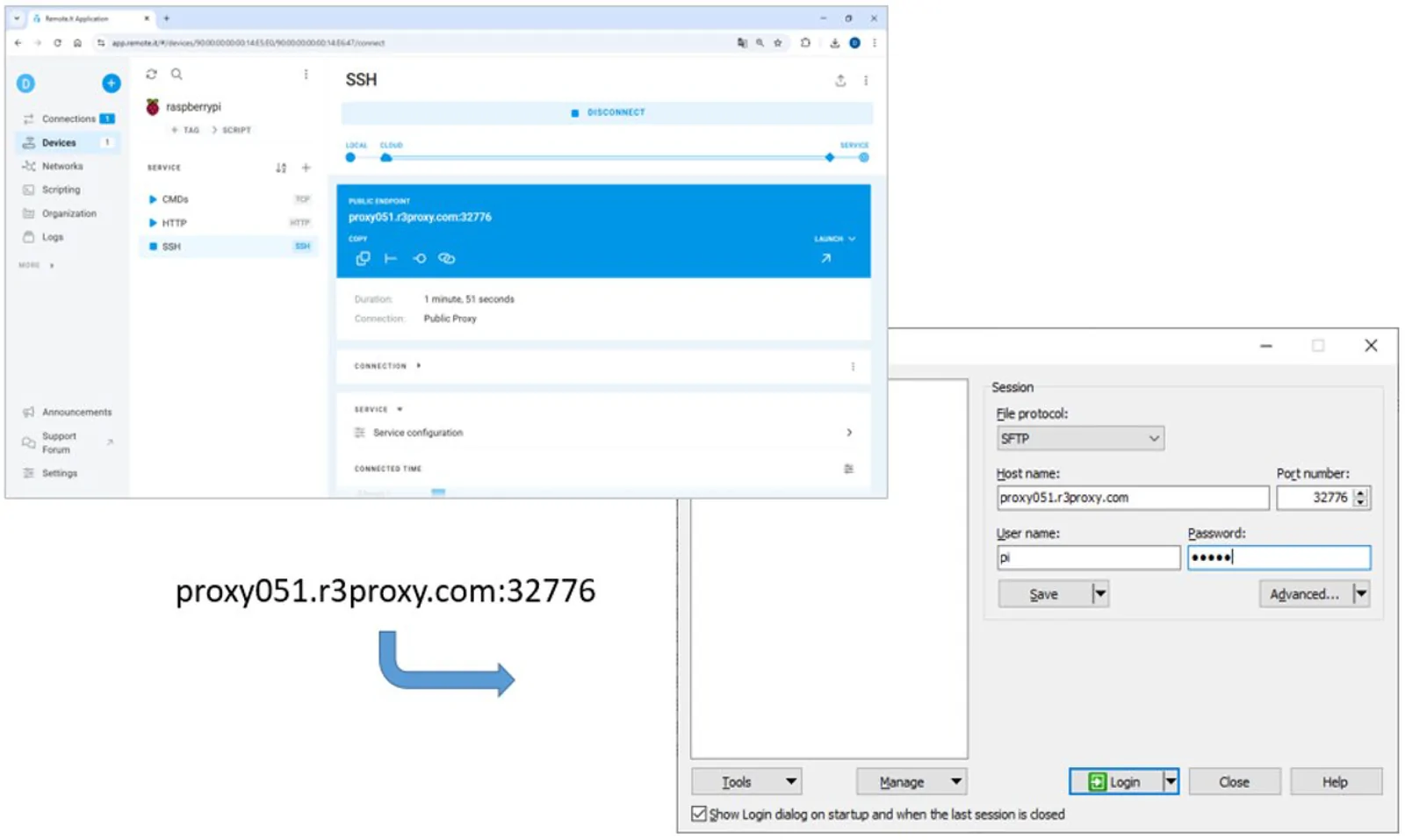
Extraction of a VPN connection identity through the Remote.It environment and its utilization for the establishment of a popular SSH-based service.

Depending on the link between the farm and the rest of the world, a proper suite of services can be installed ensuring continuous data availability to scientists and farmers. More specifically, we configured and activated the Remote.It virtual private network (VPN) service to provide fertile ground for using a wide set diverse applications, varying from basic SSH console and network tools to sophisticated graphical environment packages and/or popular web-based services. Figure 8 illustrates the generation/extraction of a VPN connection identity through the Remote.It environment and its utilization the establishment of an SSH-based client-server application (i.e., the WinSCP file management suite).

Seeking lightweight alternative implementations and using non-permanent network infrastructures, initially, the receiver was chosen to be the same LoRa32u4 II board as the sender, with the addition of a SIM card. The receiver was then able to publish the data received from the LoRa network to a remote MQTT broker by using the SIM800L module for cellular Internet connectivity. In this setup, the LoRa32u4 II board was used to handle reception of packets from the sender collars, while the SIM800L module was in charge of the cellular data connection.

However, in testing it became clear that the receiver workload was too much for one LoRa32u4 II board to handle. The board had to deal with LoRa communication, serial communication and cellular/MQTT publishing process. Therefore, the receiver architecture was split into two processing blocks assigned to different processing units. More specifically, the LoRa32u4 II board took care of the LoRa-related tasks, i.e., receiving packets from the sender collars. The received data were then sent via a serial connection to the second minimal computer board which handled the Internet and the MQTT layer. As such, the second board, the Raspberry Pi Pico microcontroller (based on the RP2040 chip), was the optimal solution, providing cost-effectiveness and satisfactory embedded processing/memory potential to receive the LoRa packet payload from the LoRa32u4 II board and to instruct the SIM800L module to publish the data toward the MQTT broker.

The whole communication chain is described as follows: first, the sender collar gathers sensor data from the animal. Second, the LoRa32u4 II board formats the collected values and sends them over LoRa. Third, the packet is received by the receiver board LoRa32u4 II and sent via serial communication to the Raspberry Pi Pico. Fourth, the Raspberry Pi Pico is used to control the SIM800L module and to establish the cellular data connection. Finally, the processed payload is published to the MQTT broker, from where it can be stored, visualized or further analyzed. This modular communication chain offers more flexibility as each subsystem is in charge of a given task: sensing and local acquisition, LoRa transmission, gateway processing, cellular connectivity, and cloud publication.

We chose the MQTT protocol for communicating with the cloud as it is lightweight and ideal for IoT applications where bandwidth is limited and connectivity is intermittent. The workflow done by the receiver was to capture the LoRa packets, prepare the payload, establish the cellular connectivity using the SIM800L module, and publish the data to a dedicated MQTT broker. The broker then became a key point for data exchange, passing the collar information to server-side applications, databases, dashboards or users.

It should be mentioned, however, that the MQTT protocol can be installed upon the bigger and more powerful Raspberry Pi unit along with the cellular functionality (via a SIM-compatible module or a suitable router). Nevertheless, the focus of this work remains on highlighting the following two extreme receiver node configurations: Raspberry Pi plus a permanent network connection and Raspberry Pi Pico plus a minimal cellular link. Both designs separate the Internet-facing infrastructure from the local LoRa network, so the farm-side gateway can operate as an edge node, with the Raspberry Pi providing more intermediate storage, processing and privacy options.

### 3.3. Power Management Issues

During implementation, a number of power-management issues should be addressed. The requirement for long-time continuous operation on batteries is important. For this reason, optimization strategies were implemented at the software level, including duty cycling and low-power sleep modes. The alternations between inactive and active states significantly reduce overall energy consumption while maintaining sufficient temporal resolution for monitoring. In this regard, the wearable collars continuously (in a periodic manner) collect data related to animal activity, temperature, battery status, and communication signal characteristics.

Initially, a hardware-based sleep approach was first tested with a SparkFun Nano Power Timer TPL5110 module, aiming to switch off the SIM800L module during the inactive periods and wake it up periodically every 5, 10 or 15 min depending on the reporting interval required. However, the limitations in the adjustment of the timing intervals and the increased additional wiring complexity resulted in abandoning the external hardware-timer approach in favor of a software-controlled sleep strategy, implemented by the LoRa32u4 board itself. In this way, the system both reduced energy consumption and improved control over the scheduling of measurement and transmission processes.

**Figure 9:**
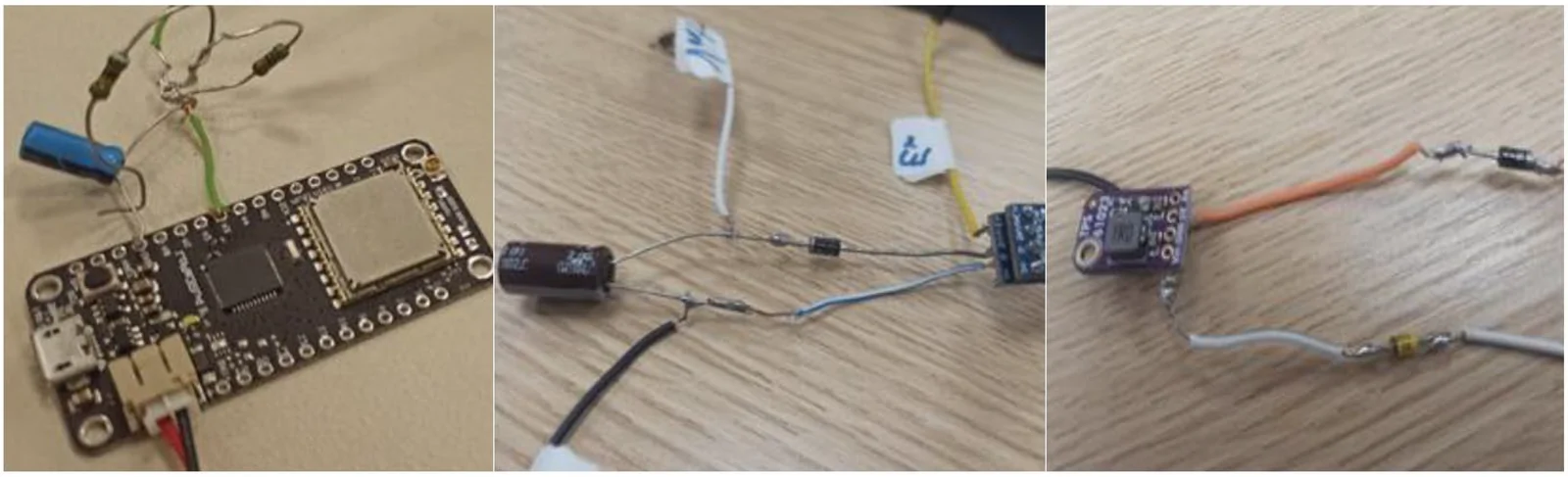
Evaluation of alternative power supply configurations and their impact on system stability, using additional capacitors, diodes and step-up converters.

A key challenge in the system design was to ensure stable power delivery to all components, particularly during wireless communication, which introduces transient current peaks. When utilizing non-permanent network infrastructures, especially for the receiving node, the most difficult part was the SIM800L module as it needs stable voltage and a high peak current to register with the network and transmit data. Several configurations were evaluated to address voltage regulation and current supply limitations. These included the use of step-up converters and alternative power regulation schemes; however, some configurations resulted in unstable operation due to insufficient current delivery under peak load conditions. The experimental configurations evaluated during development are illustrated in Figure 9.

During tests with a 3.6 V battery, the voltage at the SIM800L module was around 3 V, which is not enough to send MQTT messages reliably. The module required ~3.8–4.4 V and could draw more than 1 A, at maximum. This resulted in the receiver being unable to publish data reliably when the power supply was not properly rated for this load. The best solution was to use separate sources for the two receiver subsystems and only keep a common ground reference. This separation decreased the voltage drops caused by the high transient current of the cellular module and enhanced the overall reliability of the gateway device pair.

This experimentation indicated that the high power consumption of a typical cellular card, like the SIM800L, combined with the time (of a few seconds) required for the corresponding connection establishment with a specific cellular network provider, making it a poor choice for the mobile collar nodes themselves. On the other hand, it could remain a valuable component for a minimalistic receiver node implementation.

The final configuration employed only the stable power configuration variants, capable of supporting both sensing and communication modules, without voltage drops or system resets.

## 4. Results and Evaluation

### 4.1. Experimetal Verification Arrangments

**Figure 10:**
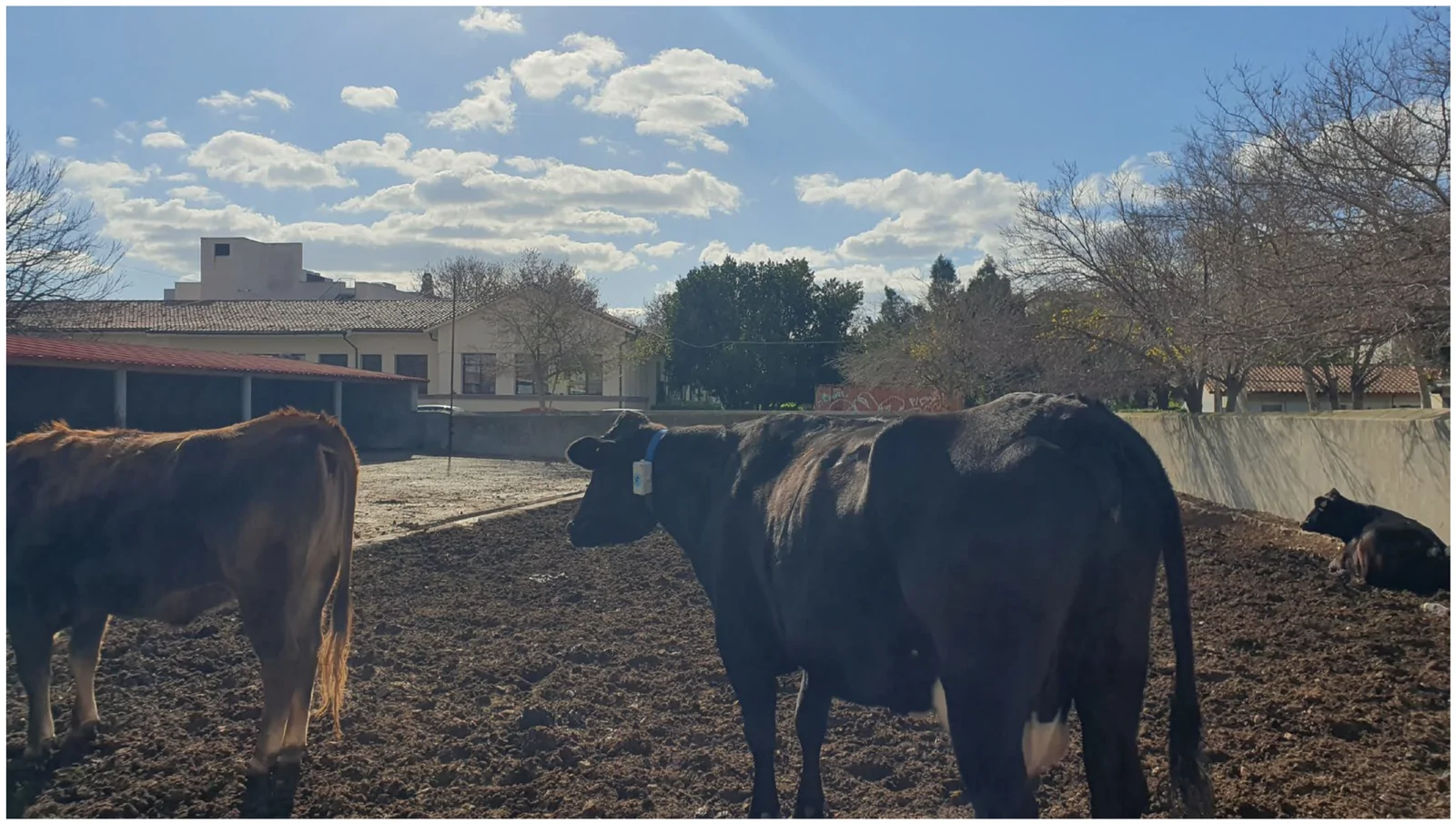
View of the livestock farm premises of the Agricultural University of Athens where the field deployment was conducted. The monitored group consisted of approximately ten cows, managed under a semi-extensive production system, four of which were equipped with the developed wearable sensor collars.

The proposed wearable collar system was deployed under real farm conditions and operated continuously over a three-month period. The experimental campaign aimed to evaluate the system’s sensing performance, wireless communication reliability, power consumption, and long-term operational stability under routine livestock management conditions. The experimental area included both the housing facilities and the outdoor exercise area used by the semi-extensively managed herd, within which the LoRa communication system was evaluated. A representative view of the study site and the animal area that was monitored is presented in Figure 10.

The sink/gateway node was placed 150 m, approximately, from the animals inside the farm premises. The system demonstrated stable operation throughout the deployment period. Data were successfully collected and transmitted without critical failures, and the device maintained consistent performance even after power interruptions. The implemented power management strategy enabled multi-day operation, confirming the effectiveness of duty cycling and low-power design.

To assess the practical applicability of the proposed platform for large-scale livestock monitoring, an indicative cost analysis of the wearable collar and gateway hardware is presented below.

The cost of purchasing and installing the collar equipment includes EUR 25 for the LoRa32u4 development board, EUR 10 required for the IMU sensor, EUR 10 for the temperature sensor and its housing, EUR 10 for the Li-Ion batteries, EUR 10 for the plastic enclosure box, EUR 15 for the fixing belt (around the neck of the animal), and roughly EUR 10 for the rest of the tinkering components, thus summing to EUR 90. A typical sink/gateway node configuration included EUR 45 for a Raspberry Pi, EUR 25 for the LoRa32u4 board, EUR 5 for an antenna, EUR 5 for the RTC module, EUR 10 for a plastic enclosure box, and EUR 35 for the power-supplying part including two 18650 batteries, resulting in a total cost close to EUR 125 for this node type. The addition of a cellular modem attachment or a 3G router was not necessary for the basic configuration setup utilizing the Raspberry Pi and fixed communication infrastructures near the farm. Regarding the Raspberry Pico and the SIM800L card scenario, the costs of EUR 5 and EUR 15, respectively, would modify the total cost above.

### 4.2. Technical Performance

The system was developed and implemented through an iterative R&D process, in which different communication, processing, power-management and cloud-connectivity configurations were implemented and evaluated.

The energy footprint of the collar nodes is a key parameter with impact on the longevity and the maintenance characteristics of the proposed system. In this regard, the LoRa 32u4 boards were equipped with a voltage divider for intercepting and reporting the voltage of the batteries powering the collars. This divider was necessary as the digitizer of the microcontroller has a native upper limit of 3.3 V, while the batteries being used may exceed the 4.0 V limit. This health status information was included inside the payload of the LoRa packets that were periodically delivered from the collars to the sink/gateway node.

**Figure 11:**
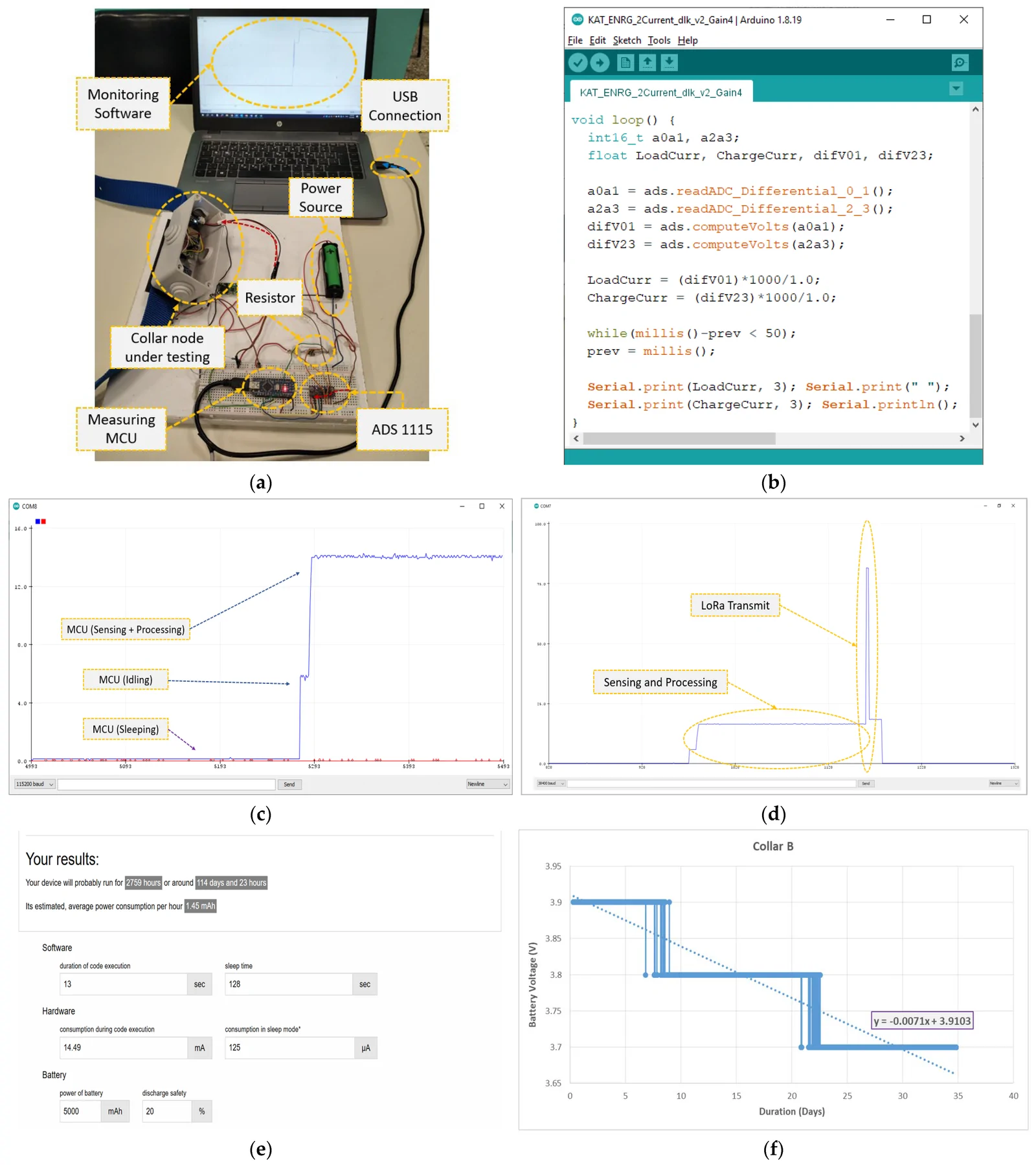
Collar node amperage consumption-related measurement arrangements and results: (**a**) the prototype real-time measuring system capturing the amperage consumption of the sensor nodes; (**b**) an indicative code part of the measuring system; (**c**) inspecting the idling, sensing/processing and sleeping collar amperage consumption via the Serial Plotter tool; (**d**) inspecting and characterizing the activity of the sensor node, in real-time, via the Serial Plotter tool; (**e**) estimation/verification of the battery life expectancy for the collars via a practical online calculator; (**f**) battery voltage drop for a collar over a period of 35 days.

Aiming to study the short-time dynamics of the sensor nodes (micro-analysis), an auxiliary measuring circuit was assembled according to the specifications in [30]. Specifically, an Arduino Nano microcontroller was utilized to calculate the voltage drops over a resistor of known value (i.e., of 1 Ω), connected in series with the sensor node inside the collar, via a separate 16-bit precision ADC module (namely, an ADS1115 unit) capable of differential measurements, connected to the Nano through its I2C interface. This real-time measuring system in action, capturing the amperage consumption of the collars, is depicted in Figure 11a, while Figure 11b illustrates an indicative part of the code running on the Nano microcontroller. The Arduino IDE environment, already used for programming the microcontrollers, favored the fast extraction of performance results and their visual representation, via the Serial Monitor and Serial Plotter components it contains. Consequently, for the node under testing, the consumption pattern could be extracted easily, with a typical time resolution close to 20 sps and an amperage resolution of 31 μA (achieved by selecting a gain factor of four for the digitizer module). The power consumption investigation focused on the behavior of the collar nodes, as typically the sink/gateway node had a dedicated small UPS-like system and its average amperage consumption (in case of RPi implementation) was close to the 300 mA level.

Delving deeper, the amperage consumption of a sensor node during sleep was close to 125 μA, while at idling it was close to 6 mA (with the LoRa radio and the unnecessary modules disabled), and while sensing and processing the IMU and temperature data were shifted to 14 mA, as shown in Figure 11c. The activity period ends with a spike corresponding to the transmission of the data over LoRa, as depicted in Figure 11d.

Continuing the power micro-analysis, by adding together the instantaneous consumption values, which were collected via the Serial Monitor tool of Arduino IDE during the activity period and dividing by the total activity duration (which was close to 13 s), an average current value close to 14.5 mA was calculated as the constant equivalent for each measurement/transmission event.

From a macro-analysis perspective, given the close to 125 μA consumption during sleep and the close to 2.5 min period of the whole process, a continuous duration close to 115 days was expected, using the online battery life estimator tool [31] for facilitating the process. The online calculator also requires defining the capacity of the battery being used (which was 5000 mAh, provided by two 18650 batteries of 2500 mAh each) and the corresponding discharge limit (which was set to 20%, indicating the remaining battery capacity); Figure 11e shows the exact parameters used and the operation duration expectancy for the aforementioned collar settings.

The above results were verified by the collar battery voltage data inside the corresponding log files that were used to form curves reflecting the voltage changes over time. Figure 11f illustrates the voltage drop (in volts) over time (in days) of the battery of a collar for 35 days and the corresponding linear trend line. More specifically, for a typical Li-Ion battery, as explained in [32], the voltage, especially for small discharge rates (i.e., that match the below 100 mA current draw of our board), drops 0.7 V approximately from the fully charged to the 80% discharged battery status. Consequently, taking into consideration the 0.0071 V/day voltage drop rate extracted by the experimental measurements depicted in Figure 11f, this indicates a battery life duration of 99 days, which is close to the results of the micro-analysis (i.e., 115 days). This small difference is justified by the approximations of the method (non-perfect linearity assumptions, truncation and rounding errors, etc.), the manufacturing deviations from the typical Li-Ion battery characteristics, and by the condition imperfections of the specific batteries being used that were not new. Added to this, the exposure to the intense outdoor temperature fluctuations also shortens battery life. Further analysis indicated that doubling the data collection interval from 2.5 min to 5.0 min, approximately, would almost double the on-battery operation duration of the collars, as the current consumption during sleep is much lower (i.e., more than 100 times) than the average activity consumption.

In all cases, the power and energy consumption equivalents could be easily extracted by the amperage data, as the collar system was supplied from a battery with a nominal voltage of 3.6 V. For instance, the node during sensing/processing consumed 50 mW, while each transmission action via LoRa was adding approximately 250 mW at 14 dBm. The LoRa transmitter was set to moderate power settings (i.e., with the coding rate set to 4/5, the bandwidth set to 128 kHz, the spreading factor set to 7, and the transmit power set to 14 dBm), thus achieving a range coverage close to 400 m. Experiments also performed with higher power settings (i.e., at 23 dBm) and commercial omnidirectional antennas (placed symmetrically inside the collar enclosures), practically achieving uninterruptible coverage close to 1000 m, in the presence of occasional obstacles such as threes and/or light farm building structures. Due to the short transmission duration of the close to 50 bytes LoRa packet payload (which was close to 100 ms) the impact of this transmit power increase to the total battery life of the collars was not significant, as it reduced this period by a few days at maximum. It must be noted, though, that the LoRa transceiver can be set to even more extreme configurations, if necessary, by altering the spreading factor (SF) and the coding rate (CR) to counterbalance noisy environments and long distances, resulting in much longer transmission times. However, this arrangement can further reduce battery duration, and it should be carefully examined against a packet retransmission policy at lower energy consumption settings.

It is worth mentioning that the multiperspective analysis being performed did not find significant differences between theoretical calculations, laboratory collar testing and in situ performance outcomes. This remark is aligned with the results of previous works of our team involving similar hardware and radio configuration settings, as the one described in Loukatos et al. [22]. Although in high interference and dense node topologies the need for retransmissions would have altered the energy requirement profile, for comparatively few nodes, elongated sleep durations and short packets (i.e., as in the farm monitoring setup being presented) did not lead to differences worth mentioning. Furthermore, the described animal activity monitoring process remains valuable even when sporadic losses are experienced, while the transmission characteristics of the small packets make the data delivery process very affordable by the battery set being adopted and not as dominant, regarding the overall energy needs during the activity period.

### 4.3. Animal-Specific Performance

**Figure 12:**
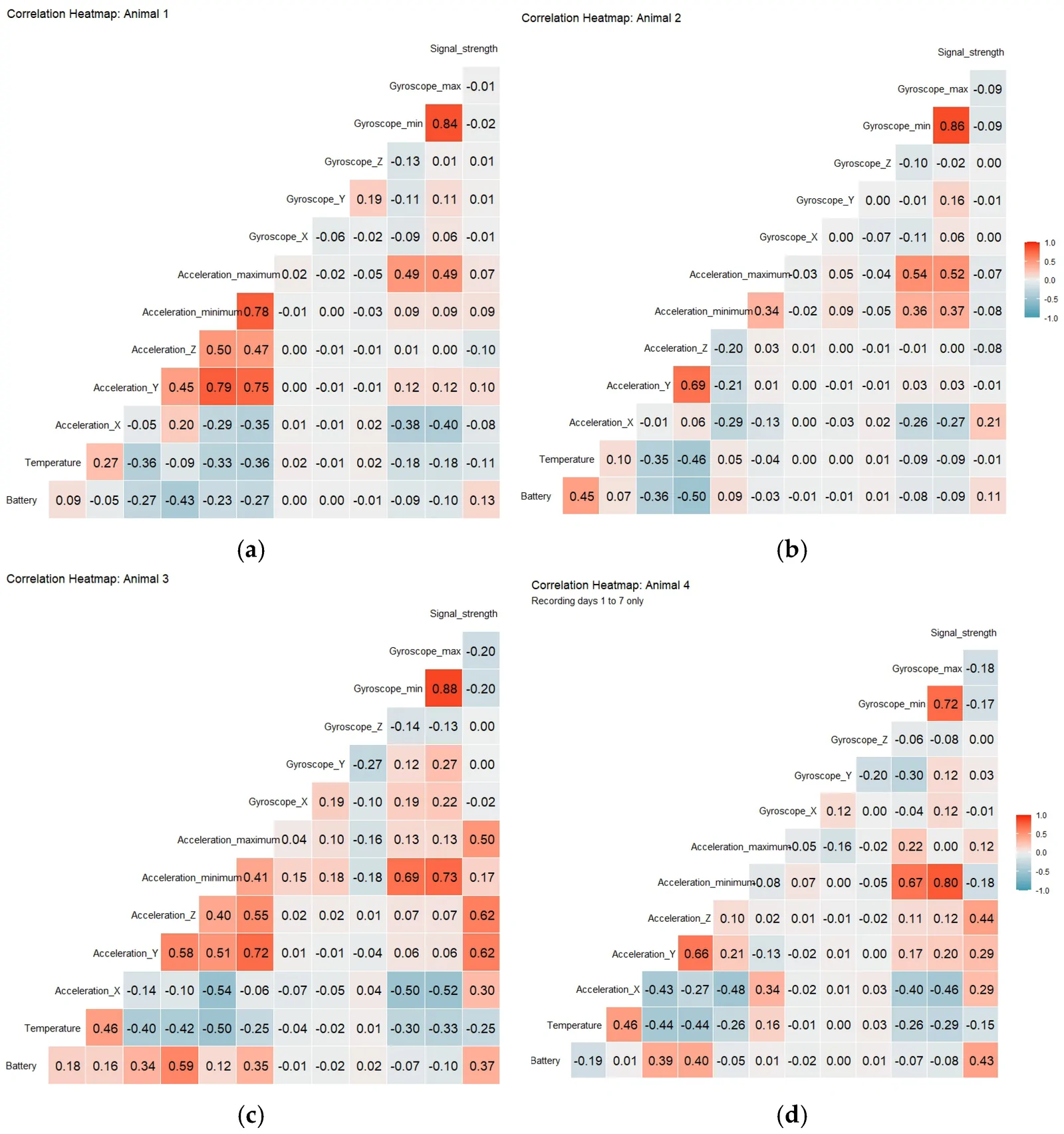
Animal-specific Pearson correlation heatmaps for collar measurements: (**a**) Animal 1, (**b**) Animal 2, (**c**) Animal 3, and (**d**) Animal 4. Variables include battery voltage, collar temperature, accelerometer-derived measurements, gyroscope-derived measurements, and RSSI. Animal 4 data from recording day 8 onward were excluded because of temperature-sensor malfunction. Red indicates positive correlations, blue indicates negative correlations, and the values within the cells represent Pearson correlation coefficients.

The data we collected consisted of accelerometer readings, temperature data, RSSI values and battery charge status. To explore relationships among the measurements obtained from each collar, correlation matrices were computed for each animal for the battery voltage, collar temperature, accelerometer-derived variables, gyroscope-derived variables, and received signal strength indicator values [33]. Correlations were computed individually for each animal to avoid mixing measurements obtained by devices with different orientation, movement patterns and deployment conditions. Data collected from recording day 8 onwards for Animal 4 were excluded due to unreliable values from the temperature sensor, because the temperature sensor exhibited a hardware malfunction that resulted in persistently unreliable measurements. The resulting correlation matrices are presented in Figure 12.

The above results basically consist of an exploratory analysis which provides insight into variable relationships and facilitates the selection and treatment of variables in sub-sequent analyses [34]. The Pearson correlation heatmaps serve as an initial preprocessing step for downstream machine learning modeling, providing insight into which variables, if used together as independent variables, will result in overfitting of the model.

The correlation matrices presented a number of common patterns in the four collars. There was a strong positive correlation between minimum and maximum gyroscope values with correlation coefficients ranging between 0.72 and 0.88. This result was expected since both variables were calculated from the same time interval of gyroscope measurements. The relationships between accelerometer variables differed between animals due to collar orientation and animal movement. Temperature was weakly to moderately correlated with the acceleration variables, but the direction and magnitude of these relationships differed between animals. Overall, the RSSI was weakly correlated with most inertial variables suggesting that short-term variation in signal strength was not simply dependent on animal activity. Some moderate correlations between RSSI and accelerometer variables were observed for individual animals, but these should be interpreted cautiously because RSSI is also affected by distance, animal orientation, obstacles, and radio propagation conditions.

**Figure 13:**
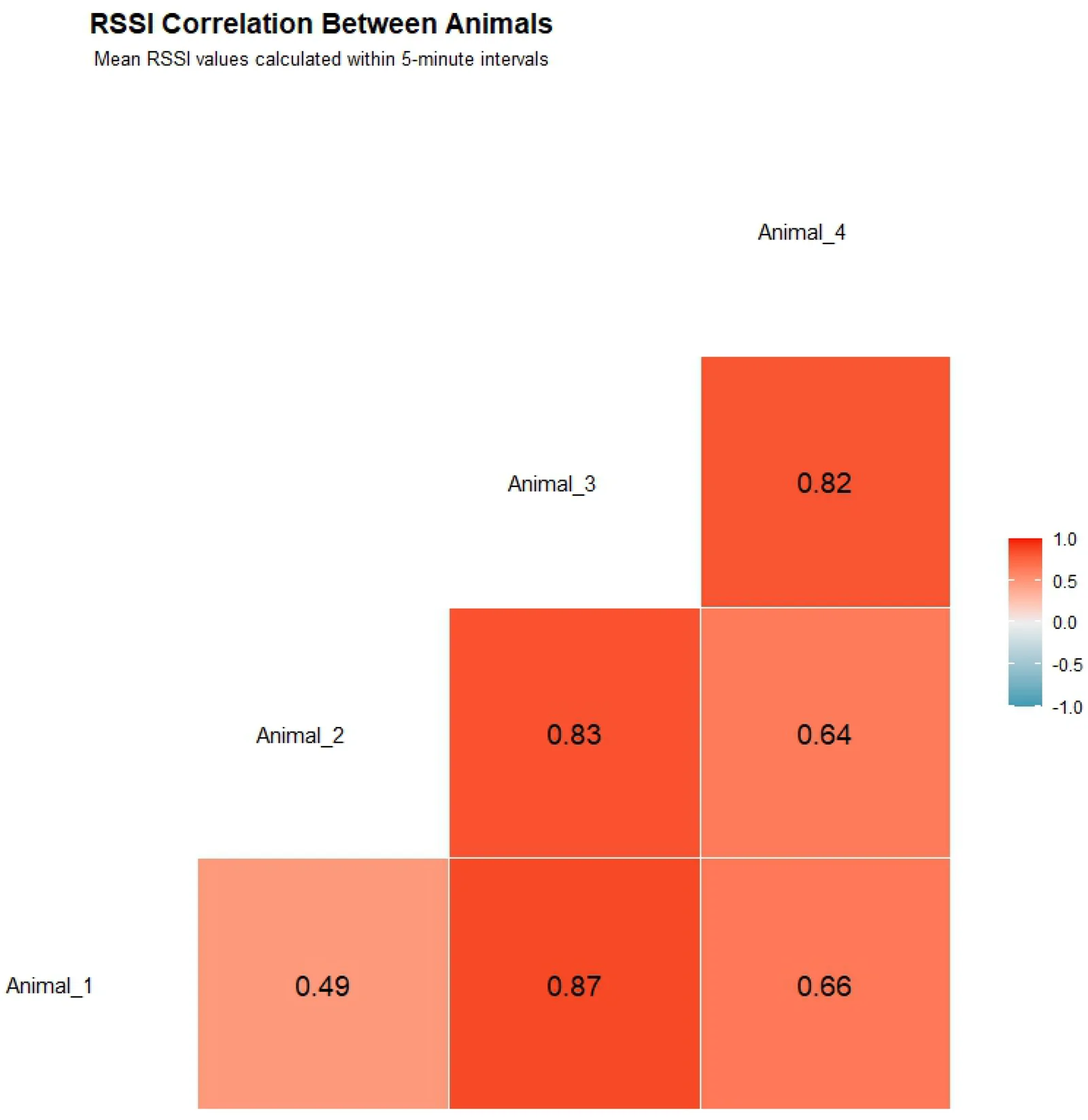
Pairwise Pearson correlations between the RSSI time series of the four collars. RSSI values were averaged within common 5 min intervals before correlation analysis. Values inside the cells represent Pearson correlation coefficients.

To determine if the collars experienced common changes in radio conditions, pairwise correlations were also calculated between the RSSI time series of the four instrumented animals. Measurements from different collars were synchronized by their acquisition time. The transmissions were not necessarily recorded at exactly the same second so observations were matched within a predefined time interval or binned into common time bins before analysis. A five-minute averaging interval was selected as a practical compromise between preserving the temporal evolution of measurements and ensuring a sufficient number of synchronized observations across collars despite the slight differences in transmission timing. For each pair of collars, Pearson correlation coefficients were then calculated, using only the time intervals that contained valid RSSI observations for both collars. Pairwise Pearson correlations between the four RSSI (Figure 13) time series showed moderate to strong positive associations. The effect of alternative temporal aggregation intervals was not investigated in the present study and will be considered in future work.

The presented correlation matrices are intended as descriptive analyses to explore relationships among the recorded variables. Formal statistical significance testing and confidence intervals were not included; therefore, the reported correlations should be interpreted as indicators of potential associations rather than evidence of causal or statistically confirmed relationships. Pearson correlation coefficients were selected because they provide a simple and widely used measure of linear association between synchronized sensor measurements. Alternative rank-based correlation measures, such as Spearman’s or Kendall’s correlation coefficients, were not investigated in the present study and constitute an interesting direction for future work.

The highest correlations were observed between Animals 1 and 3 (r = 0.87), Animals 2 and 3 (r = 0.83), and Animals 3 and 4 (r = 0.82). Moderate correlations were observed between Animals 1 and 4 (r = 0.66), Animals 2 and 4 (r = 0.64), and Animals 1 and 2 (r = 0.49). These findings indicate that part of the signal–strength variation was shared among collars, possibly because the animals experienced similar movement patterns or were exposed to common radio-propagation conditions within the monitored farm area. It is also possible that the animals exhibited synchronized behavioral responses to environmental and management conditions, such as seeking shade during periods of high temperature, clustering during colder periods, or remaining closer to water sources [35]. However, RSSI is influenced by several additional factors, including body orientation, antenna position, obstacles, gateway geometry, vegetation, and atmospheric conditions. Consequently, the observed RSSI correlations should be interpreted cautiously and should not be regarded as direct evidence of synchronized animal behavior.

Temperature readings were stable and showed the expected daily operational variations. The temperature sensor was thermally coupled to the animal through a metallic contact integrated into the collar, allowing consistent surface-contact temperature measurements throughout the deployment period. Notable differences were observed between daytime and nighttime values. These variations are compatible with the known diurnal variation in body temperature in ruminants [36]; however, because the measurements correspond to surface-contact temperature rather than core body temperature, they are also influenced by environmental conditions such as ambient temperature, solar radiation, airflow, and the quality of sensor contact. Consequently, the temperature measurements should be interpreted as a complementary indicator of the animal’s local thermal state rather than a direct physiological measurement.

The RSSI measurements varied over time as animals moved within the monitored area. Although these observations support the potential usefulness of RSSI as a complementary indicator of spatial dynamics, they should be interpreted cautiously because radio signal strength also depends on environmental and radio-propagation factors.

**Figure 14:**
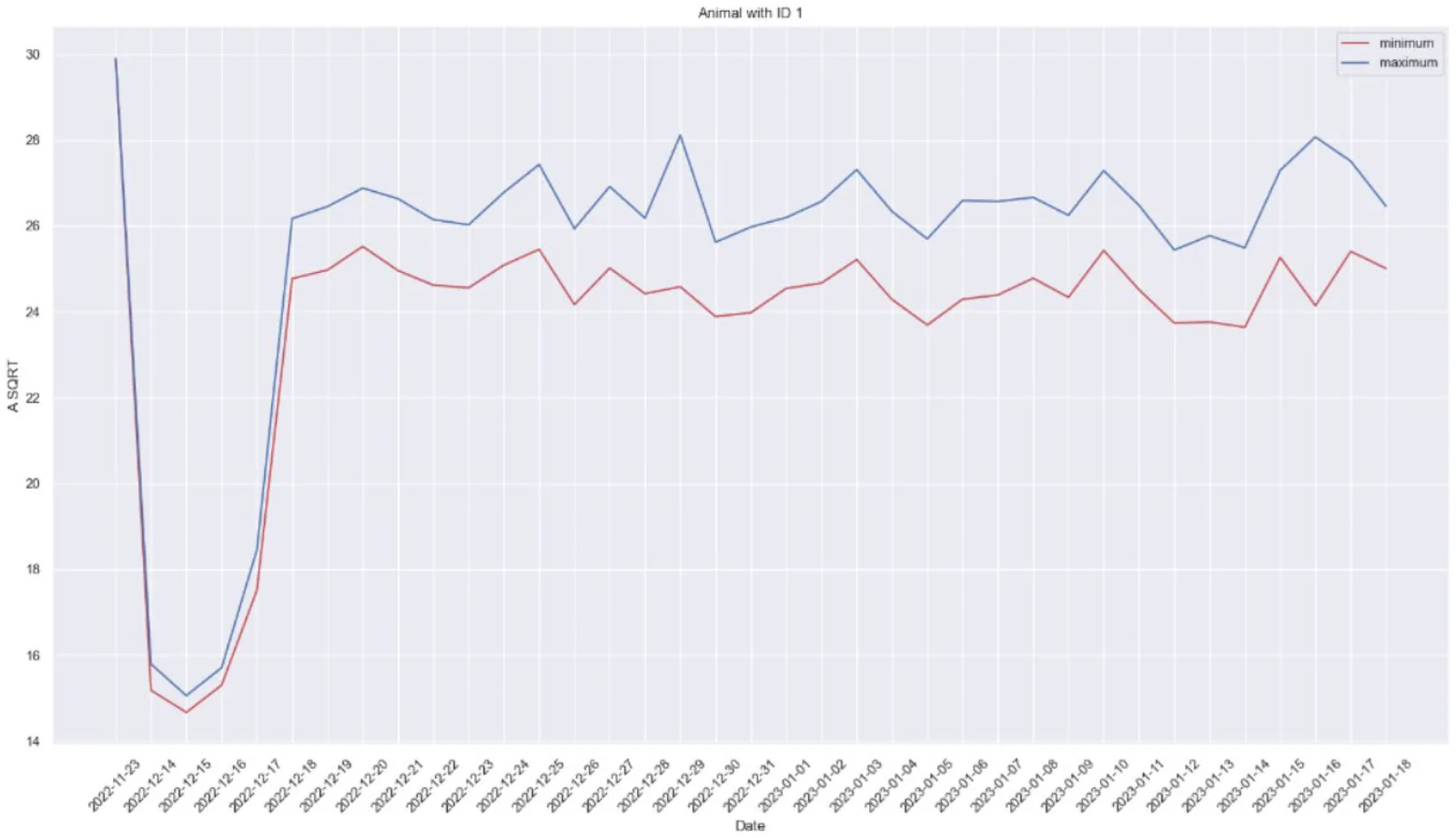
Representative accelerometer signal obtained from the wearable collar during field deployment, illustrating variations in animal activity over time.

The joint analysis of accelerometer and RSSI measurements revealed distinct signal patterns associated with differences in animal activity and relative movement within the monitored area. Figure 14 shows representative examples of the recorded sensor signals. Periods characterized by increased accelerometer variability together with relatively stable RSSI values were consistent with localized animal activity, whereas simultaneous variations in both accelerometer and RSSI measurements were indicative of broader movement within the monitored area. These observations provide qualitative insight into the recorded sensor signals but were not validated against direct behavioral observations or another reference method and should therefore not be interpreted as a formal behavioral classification.

The local storage and retransmission mechanism implemented preserved the data completeness across the experiment. Temporary communication interruptions did not result in data loss, confirming the reliability of the system under real farm conditions. The results overall confirm that the proposed wearable collar system is able to continuously, reliably and meaningfully monitor the animal activity, while keeping low power consumption and robust operation.

Figure 14 presents the consistency of sensor data over several days, proving that the system can capture consistent sensor signal patterns under real operating conditions. The observed associations between RSSI variation and relative movement, together with the relationship between accelerometer variability and activity level, suggest that meaningful sensor-derived information can be extracted using lightweight sensing and processing approaches.

### 4.4. Derived Improvements

The whole system planning, design considerations, and implementation were carried out with the aim of enabling the integration of the wearable collars with a ground station dedicated to environmental monitoring.

In this case, the gateway node would also act as a ground metering station where data on air pollution, temperature, humidity, brightness, and other environmental parameters would be collected. This makes it possible to comprehend the farm environment and, consequently, the animals’ surroundings more thoroughly. The environmental data collected will be transmitted together with the collar measurements and will be taken into consideration when interpreting animal behavior and physiological responses.

The results obtained from the present study indicate that the selected communication architecture and the low-power design of the collars provide sufficient flexibility to support such an expansion without requiring substantial modifications to the wearable devices. The usage of LoRa communication allows several remote nodes to connect with a common gateway while preserving low energy consumption and a long communication range, which are very useful in livestock farming environments.

Moreover, the combination of animal-centric measurements along with the data from the environment is expected to enhance understanding about animal behaviors and help identify possible stressors affecting animal welfare. With such an approach, it is also possible to create more sophisticated decision support systems based on correlations between animal behavior changes and environmental measurements. Thus, the proposed architectural design creates a scalable framework that can evolve into a full-fledged PLF system which will allow real-time monitoring of animals and their environment. Therefore, the proposed collar should be regarded not as the proposed collar-based architecture as a standalone wearable device, but rather as a part of the broader sensor network.

## 5. Discussion

The whole implementation and testing experience show that the proposed collar system is not merely a sensing-device assembly but a complete IoT communication-chain challenge. The collar combines sensing, embedded processing, battery operation and LoRa communication on the animal level. The receiver collects packets from many collars and acts as a local gateway at the farm level. Adding cellular connectivity and MQTT publication at the cloud level allows the data to be transferred to remote infrastructure for storage and monitoring. The development process also showed that the main practical issues were not only sensor selection related but also serial-interface limitations, power-supply stability, peak-current handling, enclosure space constraints, sleep-mode reliability and the balance between autonomy and communication frequency.

The results of this study demonstrate that low-power wearable sensing systems can provide reliable and continuous monitoring of livestock in real farm conditions with careful consideration of the system design. The proposed collar provides a compact and robust platform that successfully integrates sensing, communication and power management for long-term deployment. The practical applicability and robustness of the proposed approach was demonstrated by testing it under continuous real-farm operation, unlike most experimental systems tested under controlled conditions. The system differs from many other recent wearable animal monitoring systems by focusing more on low power consumption, modular hardware architecture, and long-term deployment rather than a variety of sensors. Such design approach aims at increasing the applicability under livestock conditions while keeping the cost of hardware low [9,29]. One of the key advantages of the system is its ability to provide objective, continuous measurements of animal activity. The traditional methods of observation are subjective by nature and are often not sensitive enough to detect early behavioral changes. In contrast, inertial sensing allows for the quantification of movement patterns and thus the detection of changes associated with health conditions such as lameness [6,11]. The results from the collected accelerometer data show that meaningful behavioral information can be extracted even with light-weight processing techniques implemented on the device itself. Collar-based feature extraction not only minimizes the volume of data transmission but also minimizes communication energy, thus retaining only essential data for behavioral studies in the process. This balance between computation and wireless communication energy consumption is increasingly being seen as a successful approach to livestock monitoring applications [29]. The system is further enhanced with the addition of temperature sensing to provide additional physiological context. This simple measurement adds an extra dimension to the understanding of the animal’s condition when taken together with activity data. Similarly, RSSI measurements, while primarily used for communication purposes, give useful indirect information about the animal’s position and movement in relation to the base station [17].

Additionally, the proposed wearable collar follows the current trend in PLF toward integrated sensing systems that combine hardware, embedded processing and continuous data acquisition to provide objective information at the individual animal level. Similar developments have shown that the practical value of wearable devices depends not only on sensor performance but also on their ability to generate reliable information that can support management decisions under commercial farming conditions [8].

The critical importance of power management in wearable livestock systems is a key finding of this work. Duty cycling and low-power operation modes were implemented to extend battery lifetime while maintaining sufficient data resolution. These strategies enable deployment practically without frequent battery replacement, which is one of the key requirements for real-world farming applications [22].

Mechanical design was also seen as a decisive factor in system performance. The enclosure design and attachment method ensured durability and stability under continuous animal movement and avoid causing discomfort or disturbing normal behavior. The field deployment emphasized the crucial role of robust construction and proper sensor placement as both factors have direct implications on data quality and system longevity [9].

LoRa communication proved efficient in providing reliable long-range communication at low energy costs. As LoRa enables communication across long ranges, LoRa requires less infrastructure than short-range wireless communications, which makes it more compatible for grazing systems and farms [25]. This is especially true in livestock environments where animals can wander over large areas and infrastructure is often limited. By enabling stable communication with low battery consumption, LoRa makes scalable monitoring systems feasible.

The integration of multiple sensing elements in the collar emphasizes the need for multimodal data collection at the device level. The multimodal sensing model provides the foundation for future sensor fusion techniques that can utilize complementary data obtained through various sensory means and thus enhance behavioral analysis and anomaly detection at farm level. By integrating motion, temperature and communication-related data, the system can achieve a more holistic understanding of animal behavior without the need for additional hardware or increased complexity of the system [36].

The main contribution of this work is the integration of low-cost hardware design, energy-aware operation and real-world validation. The ability to extract meaningful behavioral indicators from minimal sensing resources shows that practical and scalable solutions are possible without the need for complex or high-cost sensing infrastructures. This design philosophy is in line with recent end-to-end livestock monitoring approaches, which advocate the joint optimization of sensing hardware, data processing and information extraction to deliver practical decision-support tools, instead of isolated sensing devices [16]. This agrees with Berckmans’ argument that PLF systems should avoid unnecessary transmission of raw data and instead calculate useful information close to the animal or at the lowest possible system level [5]. This supports the design choice of transmitting summarized collar variables rather than continuous raw signals [19].

Although the proposed processing strategy relies on lightweight statistical descriptors extracted from short acquisition windows, this design was intentionally selected to balance information content with computational complexity, memory requirements, communication overhead, and battery lifetime. The objective of the present work was not to maximize behavioral classification accuracy but to evaluate the feasibility of a low-cost wearable platform capable of reliable long-term autonomous operation under practical farm conditions. More sophisticated edge-based approaches, including TinyML and embedded machine learning inference, have recently become feasible through the use of more powerful microcontrollers with substantially larger memory and processing resources. However, such approaches typically require greater computational complexity, larger memory footprints, and additional software development, while also affecting overall energy consumption depending on the hardware platform and inference frequency. Consequently, the adopted lightweight feature extraction strategy represents a deliberate engineering trade-off between computational efficiency, communication requirements, and operational autonomy. The selected duty cycle was similarly chosen as a compromise between temporal sampling resolution and battery lifetime, allowing approximately three months of autonomous operation while periodically capturing representative snapshots of animal activity. Future work will investigate adaptive sampling strategies and edge-based behavioral classification using newer low-power embedded platforms.

While the results demonstrate the feasibility of the proposed wearable platform under real farm conditions, several limitations should be acknowledged.

During the field deployment, one collar exhibited a hardware malfunction affecting the temperature sensor, resulting in persistently unreliable temperature measurements after the eighth recording day. Consequently, these measurements were excluded from the corresponding analysis. Although the remaining sensing, communication, and power-management functions continued to operate normally, this observation highlights the importance of long-term sensor reliability and hardware robustness under practical livestock farming conditions. Future hardware revisions will focus on further improving the reliability and durability of the sensing module during extended deployments.

Furthermore, the statistical analysis presented in this study is exploratory in nature. The use of Pearson correlation coefficients and a fixed temporal aggregation interval provide an initial assessment of variable relationships; however, future work could investigate the robustness of these findings using alternative correlation measures and different temporal aggregation strategies.

In future work, the attention should be on integrating machine learning techniques for behavior classification and anomaly detection at the edge or near the edge [37]. The system should benefit from the recent availability of end devices with higher processing and especially memory characteristics than the small 32u4 microcontroller, thus being able for elementary on-device artificial intelligence functionality, with respect to the efficient implementation of the sleep mode. Further power consumption optimization and synergies between the hardware components will also be important for improving usability and scalability. The system will be extended to larger herds and for longer periods of deployment to further validate the system under different farming conditions.

To summarize, the proposed wearable collar system shows that practical, low-cost and energy-efficient solutions can enable continuous monitoring of livestock when designed with a focus on real-world constraints. The outcomes help the development of robust wearable technologies for PLF and provide guidance for future design and deployment of systems.

## 6. Conclusions

This work presented the design considerations, implementation and validation in field of a low-power, low-cost, wearable sensor collar for continuous monitoring of ruminants under real farm conditions. The proposed system combines inertial sensing, temperature measurement and LoRa-based communication in a compact and robust wearable platform. The system design addresses practical considerations such as sensor selection and placement, enclosure design and attachment method.

The results demonstrate that reliable long-term operation can be obtained by careful hardware design and power management strategies. The implementation of duty cycling and low power modes of operation allowed for an increase in the battery lifetime with continuous data acquisition. Field deployment confirmed stable system performance, satisfactory data completeness and reliable communication under realistic conditions.

The data collected (including accelerometer, temperature and RSSI readings) provide meaningful insight into animal activity and behavior. This combination of signals helps to better interpret movement patterns without increasing the complexity of the system, demonstrating the value of lightweight, multi-sensor approaches in wearable livestock monitoring.

The real-world deployment experience indicates that tradeoffs in design must be made between power, communication reliability, mechanical robustness, and wearability. The results have practical implications for designing energy-efficient and scalable wearable sensing systems for PLF applications.

Future work will include sophisticated data analysis and machine learning methods for automated behavior classification and anomaly detection. In addition, further optimization of hardware design and system scalability for larger deployments will be investigated.

## Figures and Tables

**Table 1:** Comparison of representative wearable livestock monitoring systems with the proposed system.

Study	Animal	Sensors	Communication	Battery Life	Field Validation	Cost
[14]	cattle	Accelerometer	-	NA	No	NA
[24]	cattle	Temperature, IMU	NA	120 h	Inside barn for a few hours (10 cattle)	NA
[11]	cattle	2 IMUs: MPU9250 and BNO055, GPS	LoRa	NA	Maquehue Experimental Farm (10 dairy cows)	NA
[25]	cattle	Carbon dioxide, methane	LoRa	6 to 8 h	No	3838 THB

**Table 2:** List of data transmitted from the collar.

Variable	Description
animal_ID	Collar and animal identifier
battery voltage	Mean battery voltage from 32 ADC measurements
temp	Mean surface-contact temperature from 32 measurements
a_avg_x	Mean acceleration along the *x*-axis
a_avg_y	Mean acceleration along the *y*-axis
a_avg_z	Mean acceleration along the *z*-axis
a_sqrt_min	Magnitude formed from the minimum acceleration recorded on each axis
a_sqrt_max	Magnitude formed from the maximum acceleration recorded on each axis
g_avg_x	Mean gyroscope measurement along the *x*-axis
g_avg_y	Mean gyroscope measurement along the *y*-axis
g_avg_z	Mean gyroscope measurement along the *z*-axis
g_sqrt_min	Magnitude formed from the minimum gyroscope value recorded on each axis
g_sqrt_max	Magnitude formed from the maximum gyroscope value recorded on each axis
RSSI	Received signal strength measured by the gateway

## Data Availability

The original contributions presented in the study are included in the article; further inquiries can be directed to the corresponding author.
